# Monolayer MXene Nanoelectromechanical Piezo‐Resonators with 0.2 Zeptogram Mass Resolution

**DOI:** 10.1002/advs.202201443

**Published:** 2022-05-26

**Authors:** Dongchen Tan, Xuguang Cao, Jijie Huang, Yan Peng, Lijun Zeng, Qinglei Guo, Nan Sun, Sheng Bi, Ruonan Ji, Chengming Jiang

**Affiliations:** ^1^ Key Laboratory for Precision and Non‐traditional Machining Technology of the Ministry of Education Dalian University of Technology Dalian 116024 China; ^2^ School of Materials Engineering Purdue University West Lafayette IN 47907 USA; ^3^ Department of Material Science and Engineering Frederick Seitz Material Research Laboratory University of Illinois at Urbana‐Champaign Urbana IL 61801 USA; ^4^ Department of Physics Northwestern Polytechnical University Xi'an 710072 China

**Keywords:** functional group, mass sensor, MXene, piezoelectric, resonator

## Abstract

2D materials‐based nanoelectromechanical resonant systems with high sensitivity can precisely trace quantities of ultra‐small mass molecules and therefore are broadly applied in biological analysis, chemical sensing, and physical detection. However, conventional optical and capacitive transconductance schemes struggle to measure high‐order mode resonant effectively, which is the scientific key to further achieving higher accuracy and lower noise. In the present study, the different vibrations of monolayer Ti_3_C_2_Tx MXene piezo‐resonators are investigated, and achieve a high‐order f_2,3_ resonant mode with a ≈234.59 ± 0.05 MHz characteristic peak due to the special piezoelectrical structure of the Ti_3_C_2_Tx MXene layer. The effective measurements of signals have a low thermomechanical motion spectral density (9.66 ± 0.01 $\frac{fm}{\sqrt{Hz}}$) and an extensive dynamic range (118.49 ± 0.42 dB) with sub‐zeptograms resolution (0.22 ± 0.01 zg) at 300 K temperature and 1 atm. Furthermore, the functional groups of the Ti_3_C_2_Tx MXene with unique adsorption properties enable a high working range ratio of ≈3100 and excellent repeatability. This Ti_3_C_2_Tx MXene device demonstrates encouraging performance advancements over other nano‐resonators and will lead the related engineering applications including high‐sensitivity mass detectors.

## Graphical abstract

A monolayer MXene nanoelectromechanical piezo‐resonator is reported for the first time, achieving high‐resolution molecular sensing performance in high order mode. The effective measurements of signals have a low thermomechanical motion spectral density (9.66 ± 0.01 $\frac{fm}{\sqrt{Hz}}$) and an extensive dynamic range (118.49 ± 0.42 dB) with sub‐zeptograms resolution (0.22 ± 0.01 zg).

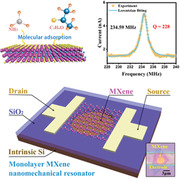

## 1 Introduction

The imperative demand for stable, sensitive, and efficient nanomechanical resonant systems has triggered research into materials, structures, and methods to perform studies for high‐sensitivity sensors and condensed matter physics.^[^ ^1^, ^2^, ^3^ ^]^ 2D materials have become the most widely used devices in mechanical resonance electronics due to the unique mechanical properties and specific surface areas of the atomic layer structures.^[^ ^4^, ^5^, ^6^ ^]^ However, the shortages of the sensitive vibration transduction and the signal acquisition technology at the atomic level remain urgent obstacles in the practical development processes.^[^ ^7^, ^8^ ^]^ It is widely believed that higher resonant frequencies, as well as the quality factor (Q‐factor), can effectively facilitate the development of resonators with enhanced signals at the atomic scale. Several monolayer materials, such as transition metal dichalcogenides (TMDCs) and graphene, are expected to achieve high operating performance due to their near‐limit surface‐to‐volume ratios and robust mechanical properties and have sparked several high‐performance resonators.^[^ ^9^, ^10^, ^11^ ^]^ However, the relatively low surface adsorption capacity and high noise hinder the nano‐resonators as ultra‐high precision detectors with complex functionalization processes.^[^ ^12^, ^13^ ^]^ For the Ti_3_C_2_Tx MXene with the high electrical performance and mechanical properties in monolayer states, the extensive presence of tunable functional groups on the Ti_3_C_2_Tx MXene provides unique features that are different from previous 2D materials, allowing more efficient adsorption of molecules as well as more sensitive ultra‐weak signals detection.^[^ ^14^ ^]^ The non‐uniformly distributed mechanical and piezoelectricity of the Ti_3_C_2_Tx MXene can potentially realize resonance signal detection of higher‐order vibrational modes at the atomic level in a resonant system.^[^ ^15^, ^16^, ^17^, ^18^ ^]^ Some of the works predict the special behavior of the MXene as a high‐performance and high‐sensitivity resonator for applications.^[^ ^19^, ^20^ ^]^ However, the exploration and implementation of the Ti_3_C_2_Tx MXene‐based nanomechanical resonance systems have not been elaborated to date yet.

Here, the resonant properties of the Ti_3_C_2_Tx MXene are investigated by constructing a high‐sensitivity piezoelectric transduction scheme based on the piezoelectric response of the asymmetric atomic structure. The high‐precision piezoelectrical method provides a more accurate resonance detection than that of conventional optical and capacitive detections. Devices driven by alternating current (AC) signals show a standard resonant waveform for the characteristic f_2,3_ resonant mode. The high‐performance molecular adsorption properties and the resonance responses of the Ti_3_C_2_Tx MXene are systematically investigated, and demonstrate ultra‐high sensitivity and reproducible multi‐molecular detection with a large dynamic range (DR). These studies report the resonance properties of the Ti_3_C_2_Tx MXene for the first time and reveal the low noise signal and high mass resolution due to the unique structure of the Ti_3_C_2_Tx MXene.

## 2 Results and Discussion

**Figure 1:**
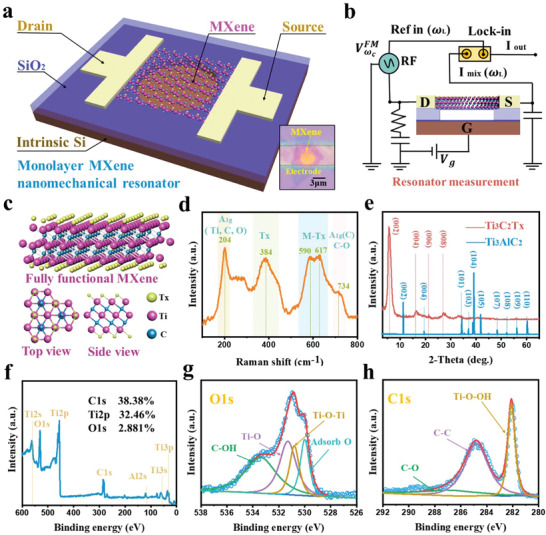
Monolayer Ti_3_C_2_Tx MXene nanoelectromechanical resonators and vibrational detection technology. a) Schematic structure of a monolayer Ti_3_C_2_Tx MXene nanoelectromechanical resonator. Right inset: the micrograph of the monolayer MXene resonator with the source and drain. b) Schematic diagram of the RF electromechanical measurement system. The DC and AC voltages are applied to the local gate and the source electrode, respectively. The mixed current I_mix_ is detected using a lock‐in amplifier and locked at the reference frequency *ω*_L_. c) 3D atomic structure of the Ti_3_C_2_Tx MXene with the top and side views. d) The Raman spectrum of the monolayer Ti_3_C_2_Tx MXene. e) XRD patterns of MAX phase (Ti_3_AlC_2_) and Ti_3_C_2_Tx MXene. f) The XPS spectrum of the Ti_3_C_2_Tx MXene, indicates the high etching purity of the material. g) O1s XPS spectrum of the Ti_3_C_2_Tx MXene, consisting of four peaks at 529.4, 530.4, 531.8, and 533.3 eV, corresponding to C–OH and Ti–O–Ti, Ti–O, and C–OH bonds. h) C1s XPS of the Ti_3_C_2_Tx MXene, consisting of three peaks at 282.2, 284.8, and 286.2 eV, representing C–Ti–OH, C–C, and C–O bonds, respectively.

The basic structure of the Ti_3_C_2_Tx MXene mechanical resonator, in **Figure** **1**a, consists of a monolayer Ti_3_C_2_Tx MXene flake on a circular‐hole vibrating cavity, source and drain electrodes, an insulating layer, and a local gate, respectively. The high‐quality synthetic Ti_3_C_2_Tx MXene can be effectively transferred to a pre‐patterned hole SiO_2_/Si substrate (The transfer method is described in Part S1, Supporting Information), and two metal electrodes (5 nm Cr and 50 nm Au) are prepared as the source and drain (the fabrication process of the Ti_3_C_2_Tx MXene resonator in Part S2, Supporting Information). The right inset of Figure 1a shows an optical micrograph of a typical monolayer Ti_3_C_2_Tx MXene resonator. As shown in Figure 1b, a designed mixing piezo‐detection technique is used to measure the vibrational frequency response. The output signal is transferred to a lower frequency without losing the stored information in its amplitude, thus achieving more convenient detection.^[^ ^7^, ^10^ ^]^ A direct current (DC) voltage is applied to the conductive Si layer as the local gate voltage to generate an electrostatic force and provides the strain to the Ti_3_C_2_Tx MXene in elastic elongation and electrostatic bias states. This AC drive power supply can also apply a frequency‐modulated voltage to detect high‐frequency mechanical motion at a lower mixed frequency, avoiding difficulties in direct radio frequency response from impedance mismatches and parasitic effects. The use of a lock‐in amplifier allows the mixed‐down current generated by the Ti_3_C_2_Tx MXene resonator as a demodulator to test the resonator amplitude at the nanometer scale with radio frequencies.^[^ ^7^ ^]^ Figure 1c presents a schematic 3D atomic structure of the fully functional monolayer Ti_3_C_2_Tx MXene. The unique multi‐atomic layer consists of the central layer and outer layer of titanium (Ti) atoms as well as the upper and lower carbon (C) atom layers encapsulated by the Ti atom layers.^[^ ^21^ ^]^ The outer layer of Ti atoms provides a large number of functional group sites to be attached.^[^ ^22^ ^]^ To fabricate the high‐quality Ti_3_C_2_Tx MXene, the minimally intensive layer delamination (MILD) method is used to prepare monolayer MXene flakes with a large‐scale flat area (specific etching strategy is described in Part S3, Supporting Information).^[^ ^23^ ^]^ Then, Raman spectroscopy, X‐ray diffraction (XRD), and X‐ray photoelectron spectroscopy (XPS) are used to characterize the quality of the synthetic monolayer Ti_3_C_2_Tx MXene, indicating the corresponding elemental composition, etching purity, and functional group composition. The Raman spectrum of the Ti_3_C_2_Tx MXene is shown in Figure 1d, in which the Raman fingerprints are located at 100 and 800 cm^−1^. The vibration at 204 cm^−1^ corresponds to the vibration A_1g_ of Ti, C, and surface functional groups, while the vibration at 734 cm^−1^ produces a redshift concerning the solution or film as the vibration A_1g_ of C atoms.^[^ ^24^ ^]^ As the XRD is shown in Figure 1e, the Ti_3_C_2_Tx MXene has a little (104) peak at ≈39°and the (002) peak of Ti_3_C_2_Tx MXene moves forward to ≈6.5°compared with Ti_3_AlC_2_ MAX phase, corresponding to a C lattice parameter of 27.1 Å, confirming the high purity of the sample.^[^ ^25^ ^]^ In Figure 1f, the surface electronic states and surface compositions of the Ti_3_C_2_Tx are analyzed by an XPS. According to the XPS spectrum, the dominated peaks of Ti and C indicate the high purity of the Ti_3_C_2_Tx MXene in the fluoride etching strategy. And in Figure 1g, the O1s XPS spectrum consists of four peaks located at 529.4, 530.4, 531.8, and 533.3 eV, corresponding to O adsorption, C–OH, and Ti–O–Ti, Ti–O, and C–OH bonds.^[^ ^26^ ^]^ In Figure 1h, C1s XPS consists of three parts, located at 282.2, 284.8, and 286.2 eV respectively, representing C–Ti–OH, C–C, and C–O bonds. The above characterizations show the successful preparation of the high‐quality Ti_3_C_2_Tx MXene samples, with the surface functional group ‐OH.^[^ ^27^ ^]^

**Figure 2:**
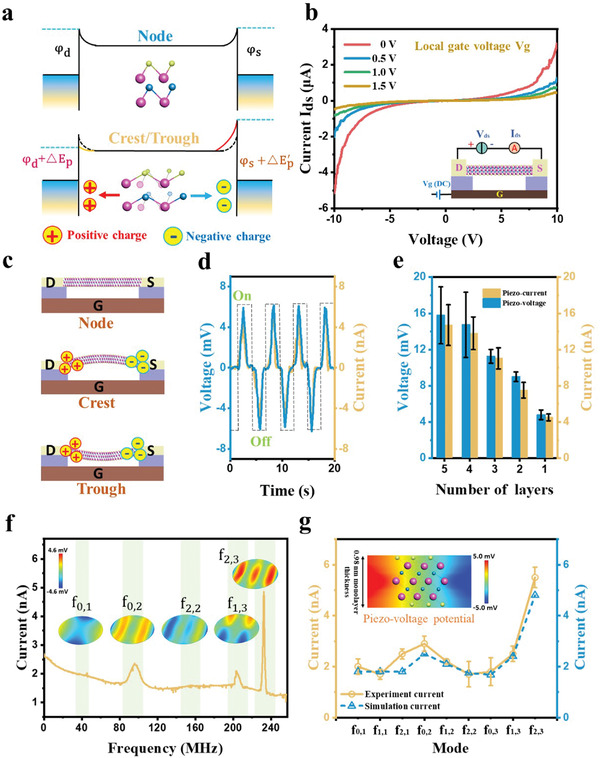
Piezoelectric modulations and resonant behaviors of the Ti_3_C_2_Tx MXene. a) Modulated barriers by piezoelectric charges due to structural deformation of Au‐MXene‐Au. *φ*_d_ and *φ*_s_ are the metal barrier heights on drain and source, respectively. Δ*E_p_* and $\Delta E_{p}^{,}$ are the corresponding changed piezo‐potentials caused by the piezoelectric polarization charges. b) *I–V* curves of the suspended monolayer Ti_3_C_2_Tx MXene with the driven gate voltage, illustrating the asymmetric modulation behavior due to the piezoelectric effect. c) The representative motion states of the monolayer Ti_3_C_2_Tx MXene resonator switch between the stretch and recovery, generating the corresponding piezoelectric polarization charges. d) The piezoelectric current and voltage signals of the monolayer Ti_3_C_2_Tx MXene under the driven AC gate voltage, illustrate the piezoelectric response process during vibration. e) Relationship between piezoelectric response current and voltage with the different layer numbers of the Ti_3_C_2_Tx MXene. f) Different resonant responses of the monolayer Ti_3_C_2_Tx MXene resonator, acquired by the sweeping frequency with the piezoelectrical transconductance system. At high order f_2,3_ resonant frequency, a prominent characteristic peak can be explicitly obtained at 234.59 MHz in the Ti_3_C_2_Tx MXene resonator. g), Relationship between measured and simulated piezo‐resonant response signals with different resonant frequencies in the monolayer MXene resonator. Inset: the particular piezo‐potential distribution in a monolayer MXene structure.

As the MXene resonator, the metal‐MXene‐metal structure allows resonant deformation to produce piezo‐electric transport characteristics through the vibrations of the Ti_3_C_2_Tx MXene.^[^ ^8^ ^]^ This modulation scheme is mainly from the piezoelectric response current based on strain‐induced polarization charges,^[^ ^28^ ^]^ and the mechanical vibration of the Ti_3_C_2_Tx MXene is variable to control the output piezoelectric electric signal.^[^ ^29^, ^30^ ^]^ For the Au‐MXene‐Au structure, the Fermi energy level is flat with an unstretched state in the upper of **Figure** **2**a and the internal electric dipole moment unit is in a balanced steady‐state. Under the deformation, the electric dipole moment is out of equilibrium, resulting in the piezoelectric polarization. Thus, the Ti_3_C_2_Tx MXene is stretched in a resonant state, while the piezoelectric polarization charge accumulates at the source and drain ends.^[^ ^15^ ^]^ These charges can raise and reduce the height of the potential barriers as the lower in Figure 2a.^[^ ^31^ ^]^ In the AC signal‐driven Ti_3_C_2_Tx MXene resonator, the resonant amplitude maintains a regular motion in response to the AC frequency and the gate DC bias. This continuous signal drives the Ti_3_C_2_Tx MXene to generate a varying piezoelectric potential, which affects the potential distribution in the resonator (The details of the piezoelectric potential modulation process are demonstrated in Part S4, Supporting Information). The deformation of the Ti_3_C_2_Tx MXene resonator induced by the DC gate voltage (the mechanical effect of the local gate capacitance on the resonator in Part S5, Supporting Information) is used to describe the transient transport characteristics during input AC signal drive, as Figure 2b for the transistor configuration. The Ti_3_C_2_Tx MXene resonator can generate a tensile strain under the action of a specific DC driving voltage (0–1.5 V). The current‐voltage (*I–V*) curve of the monolayer Ti_3_C_2_Tx MXene device produces asymmetric transmission characteristics, which are modulated by piezoelectric polarized charges from the tensile strain.^[^ ^16^ ^]^ This phenomenon is responded to the piezoelectricity as the reported works with tungstite and zincite structures, whereby piezoelectric polarization is the main cause of modulated changes in carrier transport properties.^[^ ^30^ ^]^ Furthermore, the configuration of the metal‐piezoelectric materials and the intrinsic asymmetric modulation process can introduce a transduction scheme for the strain‐induced polarization phenomenon to sense external signals. In Figure 2c, the initial state in the resonance mode is indicated, and the Ti_3_C_2_Tx MXene can be always in a cyclic strain process as stretch‐recovery‐stretch from the crest to the trough during the resonance. This process leads to the formation of the piezoelectric polarization and eventually causes the accumulated charges at the source and drain.^[^ ^8^ ^]^ Figure 2d shows the piezoelectric output in the Ti_3_C_2_Tx MXene resonator, where the responses of the piezoelectric current (short circuit) and the voltage (open circuit) are measured by a DC gate driving bias. The generation of polarized charges varies with the degree of the deformation, resulting in a piezoelectric output with the deformation cycles.^[^ ^32^, ^33^, ^34^ ^]^ When the Ti_3_C_2_Tx MXene layer is removed from its initial position and begins to stretch, the piezoelectric polarization charges accumulate and flow to the external circuit. When the layer is stretched to its limit and begins to shrink, the reversed piezoelectric polarization current flows back to form the negative response current, which directly demonstrates the conversion of the mechanical deformation to the output currents or voltages. Moreover, the experiments are also conducted on the piezoelectric output produced by different numbers of layers as shown in Figure 2e. The piezo‐output enhances with the increase of the layer number under the action of the gate voltage, the maximum open‐circuit voltage and short‐circuit current can reach 15.8 ± 2.5 mV and 14.9 ± 2.3 nA for the DC driving voltage as a 3 µm diameter circular membrane.

With the highly sensitive transduction scheme based on the piezo‐electric effect, the resonant vibrations of the Ti_3_C_2_Tx MXene layer are tested to analyze the characteristics of the system in a sealed nitrogen (N_2_) environment at room temperature (300 K). The Ti_3_C_2_Tx MXene layer is driven by an AC drive signal $V_{w_{c}}^{FM}$ with a 200‐mV source bias, causing the resonator to vibrate with a time‐varying electrostatic force at the frequency *ω*_*c*_. The Ti_3_C_2_Tx MXene resonator acts as a demodulator to trans conduct the information stored in the amplitude and generates a time‐varying piezoelectric output as a mixed current *I_mix_*, detected by the lock‐in amplifier for enabling nanoscale amplitude detection at radio frequencies.^[^ ^7^ ^]^ By scanning the driving frequency, the frequency response curve of the monolayer Ti_3_C_2_Tx MXene resonator is obtained and the multi‐response modes can be identified as shown in Figure 2f. From the frequency spectrum, the Ti_3_C_2_Tx MXene resonator can generate measurable piezoelectrical signals in the range of f_0,2_ = 90.64 MHz, f_1,3_ = 206.89 MHz, and f_2,3_ = 234.59 MHz as well as other relatively weak modes. That is because only while the piezo‐potentials are strong enough (the strain > 1.05% in the Ti_3_C_2_Tx MXene) to overcome the neutralized free electrons due to the high conductivity of the Ti_3_C_2_Tx MXene, the piezo‐signals can be effectively detected by the peripheral circuit system. The insets of Figure 2f show the simulations of each major vibration mode affecting the resonant signals, revealing the different resonant modes and the corresponding piezoelectric potential intensity. For resonant signal acquisition based on piezoelectric modulation, the piezo‐signal strength depends on the strain of the vibrational modes and the dominant distribution of the strong piezoelectric potential, as demonstrated by f_0,2_, f_1,3_, and f_2,3_. (To explain more clearly the difference in the intensity of the piezoelectric responses caused by each resonance mode, the Ti_3_C_2_Tx MXene with the piezoelectric effect is analyzed in Part S6, Supporting Information for details.) It is noticed that the f_2,3_ resonance, with the small vibrational amplitude approaching the mean free path at atmospheric pressure,^[^ ^35^ ^]^ owns a high resonance Q‐factor (Q = 228) in 1 atm. In Figure 2g, we extract the resonant response signals corresponding to the piezoelectric potentials with the simulation models. It is necessary to point out that the f_2,3_ mode exhibits an out‐of‐scale strong signal, the measured output piezo‐current has a 121 ± 4% enhancement while the corresponding strain is only 70% improvement (related to 54% piezo‐current increase)^[^ ^36^ ^]^ from f_1,3_ to f_2,3_ mode. This curious phenomenon may be caused by the special piezo‐structure of the Ti_3_C_2_Tx MXene, which leads to a non‐uniform distribution of the piezoelectric signal between the atomic layers (as shown in the inset of Figure 2g). The difference in the valence states of the inner and outer Ti atoms results in a divergence in the variation of the electric dipole moment. While resonance conditions reach a strain limit (>1.55%) of the Ti_3_C_2_Tx MXene, the strong piezoelectric charges in the inner layer produce a “leakage” effect to the surface contact region, which excites a mutational piezoelectric effect resulting in a significant enhancement of the resonant signal (Details in the Part S6, Supporting Information). Therefore, the Ti_3_C_2_Tx MXene piezo‐resonator can effectively enable the accurate extraction of a high‐order resonance mode and realize strong signal outputs with high vibration frequencies. Unlike conventional optical and capacitive measurement schemes that rely on layer vibration amplitude and signal transmission quality, the piezoelectric transconductance exploits the strong piezoelectric signal output in high‐order complex resonance modes, laying the foundation for ultra‐high precision low‐noise signal measurements.

**Figure 3:**
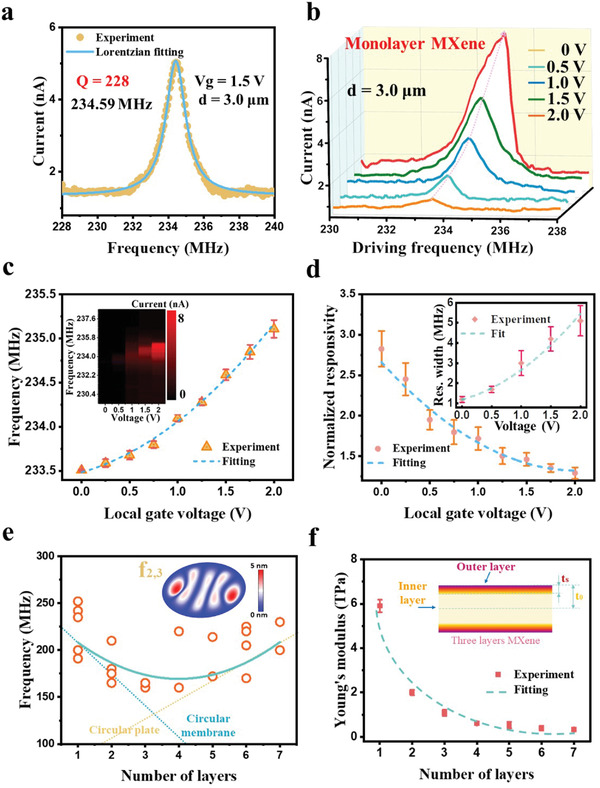
The characteristic resonances of the Ti_3_C_2_Tx MXene. a) The characteristic frequency f_2,3_ response of the monolayer MXene resonator, with the measured symmetric resonant peak signal (dots) at 234.59 MHz and the corresponding Lorentzian fit (line). b) Variation of the characteristic resonant spectrum of the monolayer Ti_3_C_2_Tx MXene resonator under different gate voltages. c) The characteristic resonant peaks as a function of the gate voltage. The inset shows the spot plot of the resonant response versus the gate voltage. d) The normalized responsivity of the monolayer Ti_3_C_2_Tx MXene resonator with the different driven voltages, and the inset shows the peak width variation under the different action of gate voltages. e) Measured characteristic resonance frequency as a function of the layer number of Ti_3_C_2_Tx MXene resonators. f) The Young's modulus of the Ti_3_C_2_Tx MXene with the different layer numbers. The inset illustrates the sandwich model of the Ti_3_C_2_Tx MXene with the inner and outer layers.

To study the basic performance of the Ti_3_C_2_Tx MXene resonators, *I_mix_* is measured as a function of the driving frequency *ω*_*c*_ for the 3 µm diameter circuit membrane resonator, as shown in **Figure** **3**a. The testing process is carried out in a sealed N_2_ atmosphere to avoid oxidation of the Ti_3_C_2_Tx MXene. The mixing current recorded for the local gate voltage *Vg* = 1.5 V shows an obvious high‐order characteristic resonance peak at 234.59 ± 0.04 MHz. At low *Vg*, the Ti_3_C_2_Tx MXene resonator receives a small driving amplitude, which is in the operating range of a linear harmonic resonator with a peak having the characteristic Lorentzian line shape and a mechanical Q‐factor estimated to be ≈228 ± 2. And the monolayer Ti_3_C_2_Tx MXene resonator achieves excellent Frequency‐Q product in f_2,3_ vibration modes as (5.25 ± 0.03) × 10^10^ *s* ^−1^. Figure 3b demonstrates a group of the typical resonant frequency response of a monolayer Ti_3_C_2_Tx MXene resonator with the 200 mV AC drive bias. The amplitude can be adjusted by changing the gate voltage, and the characteristic patterns with different gate voltages are obtained. The effect of the gate voltage on the Ti_3_C_2_Tx MXene resonator can be described as^[^ ^7^ ^]^

(1) $$F=\frac{-1}{2}\frac{\partial C_{g}}{\partial x}{\left(V_{g}-V_{sd}\right)}^{2}$$

where $\frac{\partial C_{g}}{\partial x}$ is the spatial derivative of the gate capacitance, *V_g_* is the static voltage applied to the local gate electrode, and *V_sd_* is the AC voltage between the source and drain electrodes. A larger gate voltage will result in a more intense amplitude deformation and cause a stronger piezoelectric response in the Ti_3_C_2_Tx MXene, and the detected resonant response current is able to be more pronounced.^[^ ^37^ ^]^ (Part S7, Supporting Information shows the simulation analysis of the gate voltage signal on the amplitude of the resonator.) At low gate voltage (*Vg* < 1.5 V), the resonant frequency exhibits a symmetrical peak signal, which is directly related to the pretension (T) of the circular membrane. The pretension in the *f*_2,3_ vibration mode is extracted as^[^ ^38^ ^]^

(2) $$T=6.819{\left(\frac{f_{2,3}}{b}\right)}^{2}r^{2}\rho h$$

where *ρ* is the mass density, *b* is the ratio of higher‐order modal frequency to fundamental frequency, *r* is the effective radius of the resonator, and *h* is the thickness of the Ti_3_C_2_Tx MXene layer. The pretension of the Ti_3_C_2_Tx MXene layer is estimated to be ≈0.50 ± 0.01 N m^−1^. Nanomechanical resonators with high‐aspect ratios will enter a nonlinear regime during high amplitude motion.^[^ ^39^ ^]^ As *Vg* increases to 2 V, the resonance peak changes to an asymmetric one from a symmetric line shape, confirming the Ti_3_C_2_Tx MXene resonator has become the nonlinear regime.^[^ ^40^, ^41^ ^]^ In this case, it can be described using the Duffing resonator model with an effective additional term $\frac{\alpha }{m}x^{3}$ and $\frac{\eta }{m}x^{2}\dot{x}$, which is related to the nonlinear restoring force and nonlinear damping,^[^ ^2^ ^]^ and the detailed derivation is shown in the Part S8, Supporting Information. The stable state of the Duffing equation is described as^[^ ^7^ ^]^

(3) $$\ddot{x}+\frac{k}{m}x+\left(\frac{\gamma }{m}+\frac{\eta }{m}x^{2}\right)\dot{x}+\frac{\alpha }{m}x^{3}=\frac{F_{0}}{m}cos\left(wt\right)$$

where *x* is the motional amplitude, *k* is the effective spring constant, *m* is the resonator mass, *γ* is the linear damping coefficient, *η* is the nonlinear damping coefficient, *α* is the Duffing force coefficient, *F*_0_ is the amplitude of the driving force, *ω* is the driving frequency, and *t* is time. The duffing force originates from the nonlinearities and the external electrostatic.^[^ ^42^, ^43^ ^]^ When *Vg* increases from 1.5 to 2 V, the increase in electrostatic forces acting on the Ti_3_C_2_Tx MXene resonator leads to a greater amplitude of vibration, and the Duffing force dominates the vibration process, leading to distortion of the peak shape and bi‐stability.^[^ ^39^, ^42^ ^]^ We can estimate the effective damping *γ* from the analytical solution of the linear resonant resonator equation of motion as

(4) $$Q=\frac{2\pi M_{eff}f_{2,3}}{\gamma }$$

where *M_eff_* is the theoretical effective mass of circular resonator expressed as $M_{eff}=\frac{1}{8}\pi d^{2}h\rho$ and *f*_2,3_ is the resonant frequency. The effective damping *γ* of the linear Ti_3_C_2_Tx MXene resonant resonator is estimated as $\left(9.94\pm 0.01\right)\times 10^{-10}\frac{kg}{s}$. For a circular Ti_3_C_2_Tx MXene membrane, the duffing‐type nonlinear coefficient *k* can be expressed as^[^ ^44^ ^]^

(5) $$k^{2}=\frac{13+21v-4v^{2}}{30\left(1+v\right)r^{2}\varepsilon }$$

where *v* is the Poisson's ratio of the Ti_3_C_2_Tx MXene, and *ε* is the initial strain. The critical amplitude *a_c_* determines the onset of bistability and is given by^[^ ^44^, ^45^ ^]^

(6) $$a_{c}=\sqrt{\frac{8\sqrt{3}}{9k^{2}Q}}$$

The critical amplitude of the f_2,3_ mode is estimated as 4.14 ± 0.12 nm. The thermomechanical motion spectral density at the resonant frequency f_2,3_ is given by^[^ ^44^, ^46^ ^]^

(7) $$S_{x,th}^{\frac{1}{2}}=\sqrt{\frac{4k_{B}TQ}{M_{eff}{w_{m}}^{3}}}$$

where *w_m_* is the angular resonant frequency. Based on the structure of the circular hole resonance device, the fundamental mode of thermomechanical motion out of the plane is most prominent. The thermomechanical motion is the lowest level of motion mode possible to be measured by the device and expresses the detection limit. The thermomechanical motion spectral density is defined as $S_{x,th}^{\frac{1}{2}}=9.66\pm 0.01$ $\frac{fm}{\sqrt{Hz}}$, and achieved superior performance relative to graphene,^[^ ^3^, ^39^ ^]^ molybdenum disulfide (MoS_2_),^[^ ^8^ ^]^ and black phosphorus (BP).^[^ ^47^ ^]^ The dynamic range (DR), which is defined as the ratio between the highest signal level prior to any nonlinear bifurcation and the lowest detectable level, can be described as

(8) $$DR\equiv 20log\left(\frac{0.745a_{c}}{\sqrt{2S_{x,th}\Delta f}}\right)$$

where Δ*f* is the measurement bandwidth. The DR of the monolayer Ti_3_C_2_Tx MXene resonator is 118.49 ± 0.42 dB, which is higher than the nanomechanical resonators based on the graphene,^[^ ^48^ ^]^ MoS_2_,^[^ ^49^ ^]^ and boron nitride (BN).^[^ ^50^ ^]^ The increased gate voltage leads to a DC electrostatic force between the Ti_3_C_2_Tx MXene layer and the local gate, and the generation of the additional tension lifts the resonant frequency. Therefore, the gate voltage is used to adjust the resonant frequency in atomically thin resonators.^[^ ^42^, ^51^ ^]^ The effect of the gate voltage on the resonant frequency of the monolayer Ti_3_C_2_Tx MXene resonator is indicated in Figure 3c. As the gate voltage rises, the resonant frequency shows an increasing trend. It is pointed out that the trend of resonant frequency is influenced by the built‐in strain, and the increase of the built‐in strain can make the variation curve concave, which is also consistent with the inference of pretension above.^[^ ^39^ ^]^ More simulations of this phenomenon (presented in Part S9, Supporting Information) can also illustrate the effect of gate voltage and built‐in stress on the resonant frequency. In the inset of Figure 3c, *I_mix_* versus driving frequency and the *Vg* is depicted to further analyze the effect of gate voltage on resonant response, resulting in a higher frequency response signal as well as a wider response bandwidth. The nonlinear damping on the frequency response is considered by defining the normalized responsivity as the ratio of the peak current to the drive amplitude. As shown in Figure 3d, the normalized responsivity decreases as the drive amplitude enhances, and the inset shows the gate voltage on the bandwidth of the resonant response. The wide bandwidth indicates that small changes in the different vibrational amplitudes can be amplified by the progressively increasing gate attraction, which induces a more intense piezoelectric modulation behavior.

Figure 3e illustrates the measured resonant frequency as a function of Ti_3_C_2_Tx MXene thickness (See Part S10, Supporting Information 10 for detailed analysis). The resonant frequencies of the different layer numbers in f_2,3_ modes are investigated. The resonant frequency dispersion of the same thickness can be explained by the difference in pretension as well as clamping states between the different devices.^[^ ^38^ ^]^ The statistical results show that the resonant frequencies of the Ti_3_C_2_Tx MXene resonators decrease at the beginning and then increase with the larger layer number. This phenomenon is mainly caused by the variation of the pretension and the overall Young's modulus of the layers.^[^ ^38^ ^]^ In fewer layers, the resonance characteristics are dominated by the pretension, and the contribution of Young's modulus to the resonance characteristics gradually dominates as the number of layers increases.^[^ ^10^ ^]^ This switching process from the membrane limit to the plate limit is shown by the dashed line in Figure 3e. The resonant characteristics are determined by the pretension applied to the Ti_3_C_2_Tx MXene by the resonator preparation process in the limit region of the membrane, and the frequency of *f*_2,3_ can be described as

(9) $$f_{2,3}=\frac{2.4048b}{2\pi r}\sqrt{\frac{T}{\rho h}}$$

As the thickness of the Ti_3_C_2_Tx MXene layer increases, the corresponding properties can gradually dominate and the frequency of *f*_2,3_ can be described as

(10) $$f_{2,3}=\frac{10.21b}{\pi }\sqrt{\frac{E}{3\rho {\left(1-v\right)}^{2}}}\frac{h}{4r^{2}}$$

where E is Young's modulus of the Ti_3_C_2_Tx MXene layer. As a result, the Ti_3_C_2_Tx MXene resonator exhibits different thickness dependence in the frequency response characteristics in the membrane and plate limits.

As shown in Figure 3f, for 2D Ti_3_C_2_Tx MXene with a large number of layers N (> 3), the measured Young's modulus tends to be stable at 0.38 TPa with the increase of thickness, while the measured values are dramatically increasing as N is decreasing from 3 to 1. Since high‐quality single‐crystal Ti_3_C_2_Tx MXenes with few defects are used in the experiments, it is believed that the special size dependence of Ti_3_C_2_Tx MXene with a large surface volume ratio is due to the surface modification of the material, which makes the surface effect obvious. Extensive theoretical and experimental results show that the free surface of nanomaterials can undergo a significant relaxation phenomenon characterized by bond length contraction,^[^ ^52^, ^53^, ^54^ ^]^ and the bond shrinkage can significantly affect several atomic layers below the surface of the material.^[^ ^55^, ^56^ ^]^ The elastic constants of crystals depend on the distance between the interatomic, so the surface elasticity of crystals is greatly affected by surface relaxation. A Ti_3_C_2_Tx MXene containing modified surface layers can be regarded as the composite flake shown in the inset of Figure 3f with a sandwich structure composed of an inner layer having a modulus of E_0_ and a surface layer with modulus Es which is correlated to the surface bond length contractions.^[^ ^53^, ^56^ ^]^ It is worth noting that the influence brought by the surface effect extends from the surface to the interior, gradually decreasing and eventually subsiding; however, a reasonable simplification can help in the analysis and understanding of the problem, in which the relaxed surface layer is approximately disposed of as a uniform shell. Consequently, a sandwich composite layer model is used to simplify the analysis of size‐dependent elastic properties. From the experimental results and simplified models, the repeating element with t_s_ = 0.49 nm obtained through curve fitting implies that a Ti_3_C_2_Tx MXene with one layer (0.98 nm) will be fully relaxed and have an effective modulus equal to 5.90 ± 0.28 TPa, and this model can effectively describe the elastic state from layer 1 to bulk. (See Part S11, Supporting Information for details)

**Figure 4:**
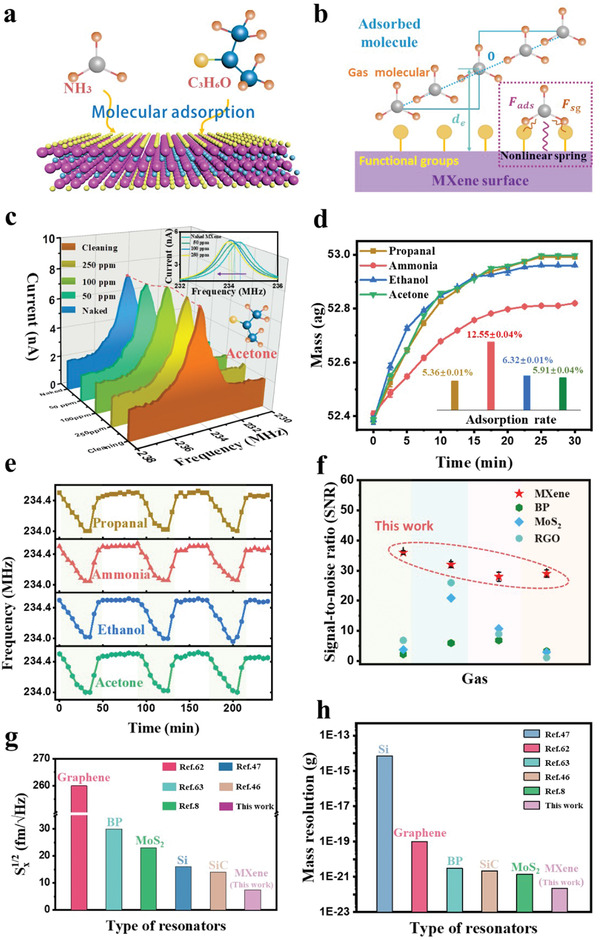
Performance of the monolayer MXene resonator as the small mass sensor. a) Schematic diagram of the adsorption process of multi‐species small molecules on a monolayer Ti_3_C_2_Tx MXene. b) The mechanism of the functional group adsorption on the surface of a monolayer Ti_3_C_2_Tx MXene. c) The shifting frequency response of the monolayer Ti_3_C_2_Tx MXene resonator with the different acetone gas concentrations. The inset shows the specific variation process of the resonant frequency peak. d) The measured different adsorption masses of acetone, propanal, ethanol, and ammonia molecules from the monolayer Ti_3_C_2_Tx MXene resonator. The inset shows the corresponding saturated adsorption rates of different gas molecules according to the surface functional group. e) Reproducibility of the monolayer Ti_3_C_2_Tx MXene resonator, and the absorbed molecules can be effectively erased with the nitrogen gas. f) Analysis of the operating signal‐to‐noise ratio of the MXene resonator as small mass detectors compared to other 2D material sensors. g) Comparison of the thermomechanical motion spectral density of the monolayer Ti_3_C_2_Tx MXene resonator and other nano‐resonators, presenting the detection limit of the Ti_3_C_2_Tx MXene system. h) The sub‐zg mass resolution of the monolayer Ti_3_C_2_Tx MXene resonator, compared with that of other nano‐resonators.

Finally, the performance of the monolayer Ti_3_C_2_Tx MXene resonator as small molecular detectors is investigated. The ‐OH functional group on the surface of the Ti_3_C_2_Tx MXene can form a hydrogen bond with the target molecule,^[^ ^14^ ^]^ resulting in stronger molecular adsorption performance than other nanomaterial resonators and affording the basis for achieving ultramicroscopic signal detection.^[^ ^14^ ^]^ As shown in **Figure** **4**a, the functional groups provide diverse molecular adsorption modalities enabling the Ti_3_C_2_Tx MXene to have good sensing properties for both organic and inorganic gas molecules. (Detailed in the Part S12 Supporting Information for the detailed mechanism, allowing multi‐species high‐performance molecular sensing properties.) As shown in Figure 4b, the equilibrium distances between the target molecules and the Ti_3_C_2_Tx MXene surfaces are able to be further reduced due to the strong adsorption of the functional groups. The inset illustrates the physical adsorption process of the target molecules on the Ti_3_C_2_Tx MXene surface. The adsorption force can be decomposed into the interaction force F_sg_ of the functional groups and the main adsorption force F_ads_ of the Ti atoms and equated to virtual nonlinear spring action. The adsorption potential energy U can be expressed as

(11) $$U=\frac{-2E_{a}d_{0}^{6}}{d^{6}}+\frac{-2E_{a}d_{0}^{12}}{d^{12}}$$

where *E_a_* is the adsorption energy of the target molecules on the Ti_3_C_2_Tx MXene surface and *d*_0_ is the adsorption equilibrium distance of the target molecules on the Ti_3_C_2_Tx MXene surface. The molecular interaction force *F_U_* with Ti_3_C_2_Tx MXene can be used to describe the ease of adsorption of molecules

(12) $$F_{U}=\frac{dU}{dd}$$

The powerful adsorption of the Ti_3_C_2_Tx MXene for small molecules (−1.2 × 10^−8^N with acetone) far exceeds that of graphene (−1.96 × 10^−9^ N with acetone),^[^ ^57^ ^]^ MoS_2_ (−1.26 × 10^−9^ N with acetone),^[^ ^14^ ^]^ and BP (−2.12 × 10^−10^ N with acetone),^[^ ^14^ ^]^ providing great potential for small molecule signal detection applications (The details of the Ti_3_C_2_Tx MXene's adsorption performance for different target molecules and comparison with other materials are provided in Part S13, Supporting Information). In Figure 4c, the resonant frequencies are tested at room temperature (300 K) in a sealed chamber filled with N_2_ by introducing acetone gas at 50, 100, and 250 ppm for 10 min. The inset shows the shift of the resonant peak frequency with increasing concentration, indicating that the adsorbed molecule number on the Ti_3_C_2_Tx MXene resonator changes with the different concentrations. The homogeneous adsorption of the gas molecules on the Ti_3_C_2_Tx MXene layer causes a change in the overall density of the layer, from which the adsorption mass variation and the number of adsorbed molecules can be obtained. For the monolayer Ti_3_C_2_Tx MXene resonator, the adsorption mass can be expressed as

(13) $$m=0.723\frac{Tb^{2}}{\pi f_{2,3}}$$

With the adsorption of molecules, ≈0.64 ± 0.01 ag acetone molecules (at 250 ppm) are able to be adsorbed on one 3 µm diameter Ti_3_C_2_Tx MXene resonator. The effects of propanal, ammonia, ethanol, and acetone on the resonant frequencies are also tested at 100 ppm concentrations as shown in Figure 4d. The masses of the adsorption molecules increase with time and reach a stable value at ≈20 min. The adsorption quantities of the corresponding target molecules are obtained from the adsorption masses, which are ≈ (6.63 ± 0.01) × 10^3^ acetone molecules, ≈ (6.63 ± 0.11) × 10^3^ propanal molecules, ≈(7.54 ± 0.08) × 10^3^ ethanol molecules, and ≈(1.42 ± 0.14) × 10^4^ ammonia molecules. It is believed that this quantitative difference stems from target molecules adsorbing to the Ti_3_C_2_Tx MXene surface in different ways, with a single molecule occupying about three attachment sites for acetone, propanal, and ethanol, and about one attachment site for a single ammonia molecule (Detailed analysis of the attachment mode of the target molecule is described in Part S14, Supporting Information). The maximum adsorption rates of four kinds of gas molecules on the Ti_3_C_2_Tx MXene surface are obtained as shown in the inset of Figure 4d, with an ammonia adsorption rate up to 12.55 ± 0.04%, propanal, acetone, and ethanol adsorption rates of 5.36 ± 0.01%, 6.32 ± 0.01%, and 5.91 ± 0.04%, respectively. The distinction with the adhesiveness of molecules has promising applications in molecular screening, molecular detection, and other fields. In Figure 4e, the Ti_3_C_2_Tx MXene mechanical resonator exhibit stable reusability, and the frequency response characteristics of the resonator are tested cyclically for propanal, ammonia, ethanol, and acetone with a concentration of 100 ppm. During the adsorption phase, the resonant frequencies decrease with time and reach stable adsorption states. Subsequently, when the target gas molecules on the Ti_3_C_2_Tx MXene surface are removed from the N_2_ surrounding, the resonators can return to the initial resonant frequencies as a repeating process.

As a small molecular detector, the signal‐to‐noise ratio is an important index to evaluate its performance, and the goal of the low interference signal is achieved by using the self‐excellent filtering effect on noise signals from the high conductivity and mixing detection techniques (The details are provided in Part S18, Supporting Information). In the f_2,3_ resonant mode, the high response signal achieves high‐quality extraction of target information, and a high signal‐to‐noise ratio of > 35 is achieved in all kinds of detection processes. As shown in Figure 4f, the Ti_3_C_2_Tx MXene nanomechanical resonator has the performance advantage of ultra‐high signal‐to‐noise ratio compared to conventional 2D material‐based gas sensors such as BP, MoS_2,_ and graphene oxide (RGO).^[^ ^14^, ^58^, ^59^, ^60^, ^61^ ^]^ Further, we compare the thermomechanical motion spectral density of various nanomechanical resonators to measure the performance of the Ti_3_C_2_Tx MXene nanoelectromechanical resonators in Figure 4g. Different from conventional optical and capacitive measurement methods, the mixing detection technique of the piezoelectric transconductance scheme can achieve low detection limits, which can be combined with the excellent molecular adsorption performance to provide a good basis for realizing a high‐performance, high‐sensitivity mechanical resonator in ultrasonic signal detection (See Part S15, Supporting Information for details).^[^ ^8^, ^46^, ^47^, ^62^, ^63^ ^]^ For our Ti_3_C_2_Tx MXene resonators, the mass resolution *δ*_*m*_ of the resonator can be derived as

(14) $$\delta_{m}\approx \frac{2M_{eff}}{Q}\times 10^{\frac{-DR}{20}}$$

Here, the ultra‐high mass resolution can be obtained as *δ*_*m*_ = 0.22 ± 0.01*zg* ([0.22 ± 0.01] ×10^−21^g), and the working range ratio of the saturated detectable mass to minimum mass resolution is up to ≈3100. This mass resolution is a prominent performance improvement compared to previously reported nanomechanical resonators as shown in Figure 4h, which stems from the unique functional group structure of the Ti_3_C_2_Tx MXene relative to other materials (See Part S17, Supporting Information).^[^ ^8^, ^46^, ^47^, ^62^, ^63^ ^]^ Table 1 summarizes the performance of the monolayer Ti_3_C_2_Tx MXene resonator and that of other reported nano‐resonators (including any pressure and temperature conditions). The monolayer Ti_3_C_2_Tx MXene not only exhibits the smallest mass resolution but also shows an excellent thermomechanical motion spectral density, which bodes well for the potential applications in microscopic signal detections.

**Table 1:** Performance comparison of nanomechanical resonators for small mass sensing

Materials	Type	f [MHz]	$S_{x,th}^{\frac{1}{2}}$[$\frac{fm}{\sqrt{Hz}}$]	Mass resolution [g]	Ref.
MXene	2D layer(1 atm, 300 K)	234	9.6	0.22 × 10^−21^	This work
Graphene	2D layer (1 atm, 300 K)	4.2	4120	4 × 10^−18^	^[^ ^64^ ^]^
Graphene	2D layer (1 atm, 295 K)	65	180	2 × 10^−21^	^[^ ^10^ ^]^
MoS_2_	2D layer (vacuum, 300 K)	60	125.3	4.5 × 10^−21^	^[^ ^5^ ^]^
CNT	1D nanotube (1 atm, 170 mK)	1.3	1700	3 × 10^−21^	^[^ ^65^ ^]^
ZnO	1D nanowire (1 atm, 300 K)	10	1180	10^−17^	^[^ ^66^ ^]^
Polysilicon	1D nanowire (vacuum, 300 K)	40	21	10^−19^	^[^ ^67^ ^]^
Polysilicon	2D arch‐bridge (vacuum, 300 K)	47.9	‐	1.5 × 10^−15^	^[^ ^68^ ^]^
Polysilicon	2D nanocantilever (1 atm, 300 K)	0.7	34.4	6.5 × 10^−17^	^[^ ^69^ ^]^
Si Nanowire	1D nanowire (1 atm, 296 K)	294	28.5	6.61 × 10^−21^	^[^ ^70^ ^]^
Si Nanowire	1D nanowire (1 atm, 300 K)	7.8	9600	2.5 × 10^−17^	^[^ ^71^ ^]^
SiC	2D nanocantilever (1 atm, 300 K)	127	39	0.7 × 10^−21^	^[^ ^35^ ^]^

## 3 Conclusion

In conclusion, the high order resonance characteristics of the monolayer Ti_3_C_2_Tx MXene nanoelectromechanical resonator are realized by the piezoelectric effect and the mixing detection technique. The atomically thin resonator exhibits a strong characteristic f_2,3_ mode response signal in the high‐frequency range, which is attributed to the special piezoelectric distribution of the 2D Ti_3_C_2_Tx MXene layer with low noise and large dynamic range. In addition, the functional groups of the Ti_3_C_2_Tx MXene play important roles in the sensing detection of target molecules with high sensitivity and large working range, revealing the working mechanism and contribution of atomic structures in molecular adsorption and low mass resolution sensing performance. This study can demonstrate the promise of the Ti_3_C_2_Tx MXene for chemical, biological and scanned‐probe sensing.

## 4 Experimental Section

### Fabrication and Integration

The preparation of the monolayer Ti_3_C_2_Tx MXene resonator consists of two parts: monolayer MXene transfer and electrode preparation. PDMS is used to transfer the monolayer MXene to the prefabricated cavity, and the morphology is confirmed by optical microscopy and atomic force microscopy (AFM). Photoresist (S1818, 5000 RPM, 45 s) is rotated on Si wafers with SiO_2_ oxide layer and pre‐baked at 115 ℃ for 1 min. The mask is aligned with the monolayer Ti_3_C_2_Tx MXene for electrode exposure, and the samples are rinsed with the developer and deionized water for 60 and 60 s, respectively. The samples are calcined at 90 °C for 1 min. Standard electrodes (5 nm Cr and 50 nm Au) are prepared by physical vapor deposition (PVD) to remove excess photoresist and metal layers. Connect external wires and seal the unit with a nitrogen‐filled chamber. See Parts S1 and S2, Supporting Information for details.

### Mechanical Detection and Actuation

A designed mixing piezo‐detection technique is used to measure the vibrational frequency response. The output signal is transferred to a lower frequency without losing the stored information in its amplitude, thus achieving more convenient detection. A DC voltage is applied to the conductive Si layer as the local gate voltage to generate an electrostatic force and provides the strain to the Ti_3_C_2_Tx MXene in elastic elongation and electrostatic bias states. This AC drive power supply can also apply a frequency‐modulated voltage to detect high‐frequency mechanical motion at a lower mixed frequency, avoiding difficulties in direct radio frequency response from impedance mismatches and parasitic effects. The use of a lock‐in amplifier allows the mixed‐down current generated by the Ti_3_C_2_Tx MXene resonator as a demodulator to test the resonator amplitude at the nanometer scale with radio frequencies.

### Statistical Analysis

The following steps are conducted for data preprocessing. Before starting the experimental steps, the sample quality is checked to obtain valid raw data. Origin software is used for statistical analysis and graphical representations of data. Data are expressed as mean ± standard error and come from three independent sets of experimental results. One‐way and two‐way analysis of variance (ANOVA) Tukey's test is used to examine the frequency distribution of different layers of MXene resonators. A *p* < 0.05 is considered statistically significant. To further investigate the signal noise, the Allan deviation is used to describe the current noise at half of the maximum amplitude (Part S18, Supporting Information). The sample size (*n*) for each statistical analysis is detailed in the legend. Statistical analysis is performed using MATLAB.

## Conflict of Interest

The authors declare no conflict of interest.

## Supporting information

Supporting Information

## Data Availability

The data that support the findings of this study are available from the corresponding author upon reasonable request.

## References

[1] O. Mashtalir , M. Naguib , V. N. Mochalin , Y. Dall'Agnese , M. Heon , M. W. Barsoum , Y. Gogotsi , Nat. Commun. 2013, 4, 1716. 23591883 10.1038/ncomms2664

[2] J. P. Mathew , R. N. Patel , A. Borah , R. Vijay , M. M. Deshmukh , Nat. Nanotechnol. 2016, 11, 747. 27294506 10.1038/nnano.2016.94

[3] C. Chen , S. Lee , V. V. Deshpande , G. Lee , M. Lekas , K. Shepard , J. Hone , Nat. Nanotechnol. 2013, 8, 923. 24240431 10.1038/nnano.2013.232

[4] K. Jensen , K. Kim , A. Zettl , Nat. Nanotechnol. 2008, 3, 533. 18772913 10.1038/nnano.2008.200

[5] J. Lee , Z. Wang , K. He , J. Shan , P. X. L. Feng , ACS Nano 2013, 7, 6086. 23738924 10.1021/nn4018872

[6] M. C. Lemme , S. Wagner , K. Lee , X. Fan , G. J. Verbiest , S. Wittmann , S. Lukas , R. J. Dolleman , F. Niklaus , H. S. J. van der Zant , G. S. Duesberg , P. G. Steeneken , Research 2020, 2020, 1. 10.34133/2020/8748602 PMC7388062 32766550

[7] S. Manzeli , D. Dumcenco , G. Migliato Marega , A. Kis , Nat. Commun. 2019, 10, 4831. 31645562 10.1038/s41467-019-12795-1 PMC6811529

[8] C. Jiang , Q. Li , J. Huang , S. Bi , R. Ji , Q. Guo , ACS Appl. Mater. Interfaces 2020, 12, 41991. 32812733 10.1021/acsami.0c11913

[9] H. Chiu , P. Hung , H. W. C. Postma , M. Bockrath , Nano Lett. 2008, 8, 4342. 19053791 10.1021/nl802181c

[10] C. Chen , S. Rosenblatt , K. I. Bolotin , W. Kalb , P. Kim , I. Kymissis , H. L. Stormer , T. F. Heinz , J. Hone , Nat. Nanotechnol. 2009, 4, 861. 19893525 10.1038/nnano.2009.267

[11] K. Hu , P. Bo , X. Li , Y. Xin , X. Bai , L. Li , W. Zhang , Europhys. Lett. 2020, 131, 58001.

[12] X. Xiao , S. C. Fan , C. Li , W. W. Xing , Sensors 2019, 19, 3027. 10.3390/s19133027 PMC6651828 31324044

[13] Z. Wang , R. Yang , P. X. Feng , Nanoscale 2021, 13, 18089. 34730595 10.1039/d1nr03286k

[14] S. J. Kim , H. Koh , C. E. Ren , O. Kwon , K. Maleski , S. Cho , B. Anasori , C. Kim , Y. Choi , J. Kim , Y. Gogotsi , H. Jung , ACS Nano 2018, 12, 986. 29368519 10.1021/acsnano.7b07460

[15] D. Tan , N. Sun , L. Chen , J. Bu , C. Jiang , ACS Appl. Nano Mater. 2021, 5, 1034.

[16] D. Tan , C. Jiang , N. Sun , J. Huang , Z. Zhang , Q. Zhang , J. Bu , S. Bi , Q. Guo , J. Song , Nano Energy 2021, 90, 106528.

[17] L. Zhang , C. Tang , C. Zhang , A. Du , Nanoscale 2020, 12, 21291. 33063799 10.1039/d0nr06609e

[18] J. Tan , Y. Wang , Z. Wang , X. He , Y. Liu , B. Wang , M. I. Katsnelson , S. Yuan , Nano Energy 2019, 65, 104058.

[19] T. Yildirim , L. Zhang , G. P. Neupane , S. Chen , J. Zhang , H. Yan , M. M. Hasan , G. Yoshikawa , Y. Lu , Nanoscale 2020, 12, 22366. 33150899 10.1039/d0nr06773c

[20] Q. Wu , W. Huang , Y. Wang , C. Wang , Z. Zheng , H. Chen , M. Zhang , H. Zhang , Adv. Opt. Mater. 2020, 8, 1900977.

[21] M. Naguib , V. N. Mochalin , M. W. Barsoum , Y. Gogotsi , Adv. Mater. 2014, 26, 992. 24357390 10.1002/adma.201304138

[22] M. R. Lukatskaya , O. Mashtalir , C. E. Ren , Y. Dall'Agnese , P. Rozier , P. L. Taberna , M. Naguib , P. Simon , M. W. Barsoum , Y. Gogotsi , Science 2013, 341, 1502. 24072919 10.1126/science.1241488

[23] M. Alhabeb , K. Maleski , B. Anasori , P. Lelyukh , L. Clark , S. Sin , Y. Gogotsi , Chem. Mater. 2017, 29, 7633.

[24] A. Sarycheva , Y. Gogotsi , Chem. Mater. 2020, 32, 3480.

[25] Q. Zhang , H. Lai , R. Fan , P. Ji , X. Fu , H. Li , ACS Nano 2021, 15, 5249. 33617227 10.1021/acsnano.0c10671

[26] K. Hantanasirisakul , Y. Gogotsi , Adv. Mater. 2018, 30, 1804779. 10.1002/adma.201804779 30450752

[27] J. Halim , K. M. Cook , M. Naguib , P. Eklund , Y. Gogotsi , J. Rosen , M. W. Barsoum , Appl. Surf. Sci. 2016, 362, 406.

[28] A. Allain , J. Kang , K. Banerjee , A. Kis , Nat. Mater. 2015, 14, 1195. 26585088 10.1038/nmat4452

[29] L. Sun , L. Zhu , C. Zhang , W. Chen , Z. Wang , Nano Energy 2021, 83, 105855.

[30] G. Hu , R. Zhou , R. Yu , L. Dong , C. Pan , Z. L. Wang , Nano Res. 2014, 7, 1083.

[31] Z. L. Wang , Nano Today 2010, 5, 540.

[32] J. He , C. L. Hsin , J. Liu , L. J. Chen , Z. L. Wang , Adv. Mater. 2007, 19, 781.

[33] Y. Gao , Z. L. Wang , Nano Lett. 2007, 7, 2499. 17645367 10.1021/nl071310j

[34] X. Wu , D. Vanderbilt , D. R. Hamann , Phys. Rev. B 2005, 72, 035105.

[35] M. Li , H. X. Tang , M. L. Roukes , Nat. Nanotechnol. 2007, 2, 114. 18654230 10.1038/nnano.2006.208

[36] W. Ma , J. Lu , B. Wan , D. Peng , Q. Xu , G. Hu , Y. Peng , C. Pan , Z. L. Wang , Adv. Mater. 2020, 32, 11905795. 10.1002/adma.201905795 31930641

[37] F. R. Ong , Z. Cui , M. A. Yurtalan , C. Vojvodin , M. Papaj , J. F. X. Orgiazzi , C. Deng , M. Bal , A. Lupascu , Nanotechnology 2015, 26, 405201. 26377034 10.1088/0957-4484/26/40/405201

[38] A. Castellanos‐Gomez , R. van Leeuwen , M. Buscema , H. S. J. van der Zant , G. A. Steele , W. J. Venstra , Adv. Mater. 2013, 25, 6719. 24123458 10.1002/adma.201303569

[39] A. Eichler , J. Moser , J. Chaste , M. Zdrojek , I. Wilson‐Rae , A. Bachtold , Nat. Nanotechnol. 2011, 6, 339. 21572430 10.1038/nnano.2011.71

[40] E. Gourdon , A. T. Savadkoohi , B. Cauvin , Build. Acoust. 2020, 27, 169.

[41] R. Liu , L. Wang , J. Appl. Phys. 2020, 128, 145105.

[42] O. Shoshani , M. I. Dykman , S. W. Shaw , Nonlinear Dyn. 2020, 99, 433.

[43] J. M. L. Miller , D. D. Shin , H. Kwon , S. W. Shaw , T. W. Kenny , Phys. Rev. Appl. 2019, 12, 044053.

[44] Z. Wang , P. X. L. Feng , Appl. Phys. Lett. 2014, 104, 103109.

[45] R. J. Dolleman , P. Belardinelli , S. Houri , H. S. J. van der Zant , F. Alijani , P. G. Steeneken , Nano Lett. 2019, 19, 1282. 30681865 10.1021/acs.nanolett.8b04862 PMC6391039

[46] Z. Wang , J. Lee , P. X. L. Feng , Nat. Commun. 2014, 5, 5158. 25399871 10.1038/ncomms6158

[47] Z. Wang , H. Jia , X. Zheng , R. Yang , Z. Wang , G. J. Ye , X. H. Chen , J. Shan , P. X. L. Feng , Nanoscale 2015, 7, 877. 25385657 10.1039/c4nr04829f

[48] J. S. Bunch , A. M. VAN DER Zande , S. S. Verbridge , I. W. Frank , D. M. Tanenbaum , J. M. Parpia , H. G. Craighead , P. L. Mceuen , Science 2007, 315, 490. 17255506 10.1126/science.1136836

[49] J. Lee , Z. Wang , K. He , R. Yang , J. Shan , P. X. L. Feng , Sci. Adv. 2018, 4, o6653. 10.1126/sciadv.aao6653 PMC5903902 29670938

[50] X. Zheng , J. Lee , P. X. L. Feng , Microsyst. Nanoeng. 2017, 3, 17038_1. 31057874 10.1038/micronano.2017.38 PMC6444998

[51] A. Keşkekler , O. Shoshani , M. Lee , H. S. J. van der Zant , P. G. Steeneken , F. Alijani , Nat. Commun. 2021, 12, 1099. 33597524 10.1038/s41467-021-21334-w PMC7889630

[52] O. Z. Didenko , G. R. Kosmambetova , P. E. Strizhak , J. Mol. Catal. A: Chem. 2011, 335, 14.

[53] Y. Y. Tay , S. Li , C. Q. Sun , P. Chen , Appl. Phys. Lett. 2006, 88, 173118.

[54] T. Li , Y. T. Li , W. W. Qin , P. P. Zhang , X. Q. Chen , X. F. Hu , W. Zhang , Nanoscale Res. Lett. 2015, 10, 394. 26450618 10.1186/s11671-015-1081-2 PMC4598335

[55] E. A. Meulenkamp , J. Phys. Chem. B 1998, 102, 7764.

[56] Y. Yang , J. Qi , W. Guo , J. Zhao , X. Wang , Y. Zhang , Appl. Phys. Lett. 2010, 96, 152101.

[57] J. Sun , M. Muruganathan , H. Mizuta , Sci. Adv. 2016, 2, 1501518. 10.1126/sciadv.1501518 PMC4846442 27152344

[58] R. Kumar , W. Zheng , X. Liu , J. Zhang , M. Kumar , Adv. Mater. Technol. 2020, 5, 1901062.

[59] Aaryashree , P. V. Shinde , A. Kumar , D. J. Late , C. S. Rout , J. Mater. Chem. C 2021, 9, 3773.

[60] S. M. Majhi , A. Mirzaei , H. W. Kim , S. S. Kim , Sensors 2021, 21, 1352.33672959

[61] G. Lee , S. Kim , S. Jung , S. Jang , J. Kim , Sens. Actuators, B 2017, 250, 569.

[62] R. M. R. Pinto , P. Brito , V. Chu , J. P. Conde , J. Microelectromech. Syst. 2019, 28, 390.

[63] M. Kumar , H. Bhaskaran , Nano Lett. 2015, 15, 2562. 25723099 10.1021/acs.nanolett.5b00129

[64] R. A. Barton , B. Ilic , A. M. van der Zande , W. S. Whitney , P. L. Mceuen , J. M. Parpia , H. G. Craighead , Nano Lett. 2011, 11, 1232. 21294522 10.1021/nl1042227

[65] S. L. de Bonis , C. Urgell , W. Yang , C. Samanta , A. Noury , J. Vergara‐Cruz , Q. Dong , Y. Jin , A. Bachtold , Nano Lett. 2018, 18, 5324. 30062893 10.1021/acs.nanolett.8b02437 PMC6089494

[66] C. Jiang , C. Tang , J. Song , Nano Lett. 2015, 15, 1128. 25575294 10.1021/nl504135x

[67] I. Ouerghi , J. Philippe , L. Duraffourg , L. Laurent , A. Testini , K. Benedetto , A. M. Charvet , V. Delaye , L. Masarotto , P. Scheiblin , C. Reita , K. Yckache , C. Ladner , W. Ludurczak , T. Ernst , in 2014 IEEE Int. Electron Devices Meeting, IEEE, San Francisco, CA 2014, 22.4.1.

[68] M. K. Zalalutdinov , J. D. Cross , J. W. Baldwin , B. R. Ilic , W. Zhou , B. H. Houston , J. M. Parpia , J. Microelectromech. Syst. 2010, 19, 807.

[69] J. Verd , G. Abadal , J. Teva , M. V. Gaudo , A. Uranga , X. Borrise , F. Campabadal , J. Esteve , E. F. Costa , F. Perez‐Murano , Z. J. Davis , E. Forsen , A. Boisen , N. Barniol , J. Microelectromech. Syst. 2005, 14, 508.

[70] J. Molina , J. E. Escobar , D. Ramos , E. Gil‐Santos , J. J. Ruz , J. Tamayo , Á. S. Paulo , M. Calleja , Nano Lett. 2021, 21, 6617. 34288677 10.1021/acs.nanolett.1c02056 PMC8361434

[71] Y. Lu , S. Peng , D. Luo , A. Lal , 25th IEEE Int. Conf. on Micro Electro Mechanical Systems, IEEE, Paris, France 2012, 88.

