# Supplementary material for: Monolayer MXene Nanoelectromechanical Piezo‐Resonators with 0.2 Zeptogram Mass Resolution
Source: Adv Sci (Weinh). 2022 May 26;9(22):2201443. doi: 10.1002/advs.202201443 (PMC9353497; doi:10.1002/advs.202201443)
Supplement: Supplementary file 1 — Supporting Information [file ADVS-9-2201443-s001.pdf]

ADVANCED SCIENCE Open Access

Supporting Information

for *Adv. Sci.*, DOI 10.1002/advs.202201443

Monolayer MXene Nanoelectromechanical Piezo-Resonators with 0.2 Zeptogram Mass Resolution

*Dongchen Tan, Xuguang Cao, Jijie Huang, Yan Peng, Lijun Zeng, Qinglei Guo, Nan Sun, Sheng Bi, Ruonan Ji and Chengming Jiang**

Monolayer MXene nanoelectromechanical piezo-resonators with 0.2 zeptogram mass resolution

(Supporting Information)

1. The all-dry transfer method of Ti_3C_2Tx MXene-based construction device.

The conventional MXene drop-in method is not suitable for preparing high-quality suspended Ti_3C_2Tx MXene construction on a circular hole resonant cavity due to the presence of liquid, leading to surface cracks and contamination of these suspended Ti_3C_2Tx MXene flakes during the drying process. As shown in **Figure S1a**, the hydrophilic Ti_3C_2Tx MXene flakes will be dragged into the holes by significant surface tension during the evaporation of water in Ti_3C_2Tx MXene suspension. Furthermore, the violent motion of drying water molecules will accelerate the agglomeration of Ti_3C_2Tx MXene flakes and the buildup of Ti_3C_2Tx MXene groups, resulting in the inability to remove impurities of the flake surface. Therefore, the presented Ti_3C_2Tx MXene device is constructed by an all-dry transfer method. And PDMS can screen out relatively large size and good quality Ti_3C_2Tx MXene flakes without impurities, as shown in **Figure S1b**, no obvious agglomerates and wrinkles are observed on the transferred flake surface. As shown in **Figure S1c**, the principle of the all-dry transfer is that the adhesion F_p between the hydrophilic Ti_3C_2Tx MXene flake and the hydrophobic PDMS is weaker than F_s between the flake and the SiO_2 surface[1]. The transfer procedures are as follows: circular hole resonant cavities were firstly prepared on

SiO_2 layers of SiO_2/Si substrates and the Ti_3C_2Tx MXene suspension was diluted to 0.01 mg/mL. Subsequently, the PDMS stamp was immersed in this suspension for 2 seconds. Extensive Ti_3C_2Tx MXene flakes are adsorbed on the surface of PDMS and the impurities in the suspension were effectively isolated because the hydrophobic PDMS cannot adhere to the water. Finally, the PDMS with Ti_3C_2Tx MXene flakes is pressed onto the silicon wafer with circular resonant cavities slowly.

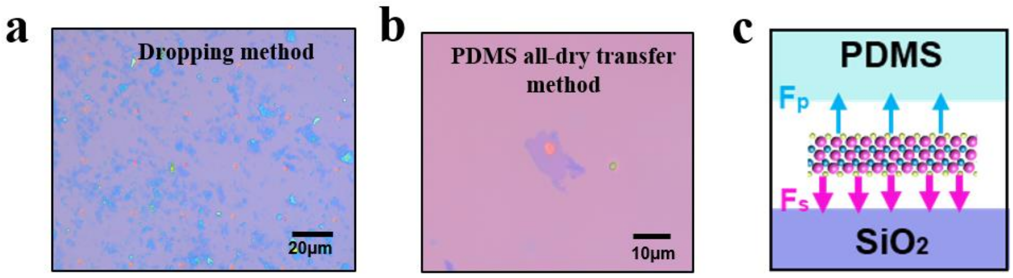

Figure S1 **a**, The formation of water stains and agglomerates of Ti_3C_2Tx MXene flakes caused by dropping method. **b**, The suspended Ti_3C_2Tx MXene flake on a resonant cavity is transferred by the PDMS-based all-dry transfer method. **c**, Principle of the all-dry transfer method.

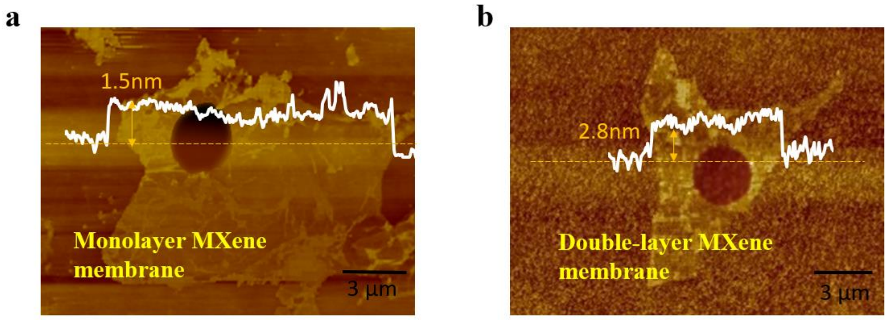

Figure S2 **a**, Single-layer Ti_3C_2Tx MXene on a resonant cavity transferred by the PDMS-based all-dry transfer method. **b**, Double-layer

Ti_3C_2Tx MXene on a resonant cavity transferred by the PDMS-based all-dry transfer method.

2. The fabrication process of the Ti_3C_2Tx MXene monolayer nanoelectromechanical piezo-resonators.

Figure S1 shows the fabrication process of the monolayer Ti_3C_2Tx MXene resonator. And the detailed fabrication steps are exhibited as follows:

1. Photoresist (S1818, 5000 RPM, 45 s) is rotated on Si wafers with SiO_2 oxide layer and pre-baked at 115 °C for 1 min.
2. The shape is exposed with a mask aligner, and the samples are washed with the developer and deionized water for 60 s and 60 s, respectively. The samples are baked at 90 °C for 1 min.
3. Reactive ion etching (RIE) is used to etch the round hole channel, and the excess photoresist is removed by acetone and deionizing water washing, and the water is removed by baking at 100 °C for 2 minutes.
4. Take a clean and flat PDMS sheet, immerse it in dilute Ti_3C_2Tx MXene aqueous solution 2 s, and take it out. Use clean paper to absorb the residual water droplets on the PDMS sheet, gently spread the PDMS sheet on the Si wafer prepared with pre-channel, gently press to make the PDMS sheet fit the silicon wafer completely without bubbles, and then slowly remove the PDMS sheet.
5. The transferred Si wafers are baked at 100°C for the 30s to remove

residual moisture. Then, the position of the successfully transferred Ti_3C_2Tx MXene is preliminarily determined by an optical microscope, and the exact thickness of the Ti_3C_2Tx MXene is determined by an atomic force microscope (AFM) to determine the number of layers.

6. The mask is aligned with the monolayer Ti_3C_2Tx MXene for electrode exposure, and the samples are washed with the developer and deionized water for 60 s and 60 s, respectively. The samples are roasted at 90 ℃ for 1 min.

7. Standard electrodes (5 nm Cr and 50 nm Au) are prepared by physical vapor deposition (PVD), and the excess photoresist and metal layer are removed by the extraction method.

8. Connect the external wire and seal the device with a nitrogen-filled chamber.

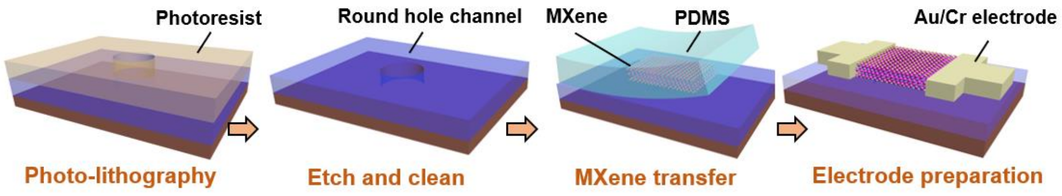

Figure S3 Fabrication process of Ti_3C_2Tx MXene resonator.

3. Etching strategy of Ti_3C_2Tx MXene.[2]

The minimum strength layer delamination etching strategy (MILD) is adopted to prepare Ti_3C_2Tx MXene, and the specific steps are as follows:

1. Weigh 4.8 g lithium fluoride (LiF) and 60 mL 9 M hydrochloric acid (HCl), mix them several times and stir them in a Teflon beaker for 5 minutes to make LiF completely dissolved in HCl. The beaker is provided with a vent hole, the reaction gas can be discharged.

2. 3.0 g Ti_3AlC_2 is weighed and slowly added into the etching solvent several times, then stirred in a magnetic stirrer at 40 ℃ for 24 hours.

3. After the reaction, centrifuge with 1M HCl 3 times to remove the residual LiF impurities, and then rinse with deionized water until the pH value of the supernatant reaches 6.0. This process needs to be repeated 5-6 times to facilitate separation.

4. The etched Ti_3C_2Tx MXene is layered by the acoustic method and handshaking method, and the final product is centrifuged at the speed of 3500 rpm for 1 hour.

4. Piezoelectric potential modulation process in Ti_3C_2Tx MXene.[3-6]

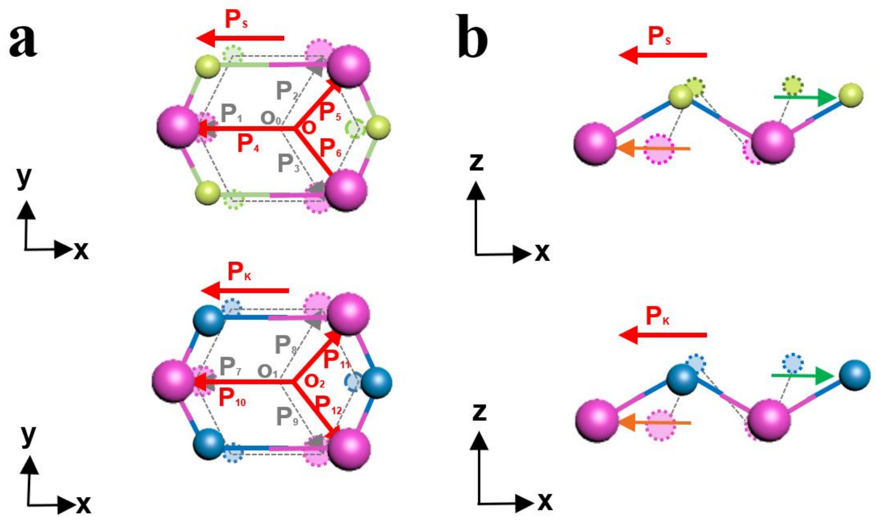

Figure S4 Piezoelectric polarization principle of Ti_3C_2Tx MXene. a, The 3D Ti_3C_2Tx MXene structure is simplified to consist of hexagonal atoms in the x-y direction of the top view, and the electric dipole moment vector in the equilibrium state is kept in equilibrium. Under the action of external force, space displacement occurs between atoms, and the electric dipole moment vector is unbalanced, resulting in piezoelectric polarization. **b**, The x–z plane exhibits the atomic composition of the side view, the transverse electric dipole moment imbalance during stretching produces piezoelectric polarization and generates a piezoelectric electric field.

As shown in **Figure S4a**, the Ti_3C_2Tx MXene is divided into 2D atoms in the x-y plane and the x-z plane, consisting of Ti-Tx and Ti-C, respectively. When subjected to deformation in the x-direction, the stretching in the

x-direction will cause the electric dipole moment of the hexagonal 2D atomic group to be unbalanced, resulting in an electric dipole moment vector in the x-direction, resulting in piezoelectric polarization. This process is reflected in the lateral deformation of the zigzag atomic composition in **Figure S4b**. Furthermore, due to the repeating units of Ti-Tx and Ti-C structures, it is plausible that Ti_3C_2Tx MXene has in-plane piezoelectric properties when subjected to external strain.

5. The mechanical effect of the local gate capacitance on the resonator.[7]

The force acting on the Ti_3C_2Tx MXene membrane by the gate can be described as

$$F = -\frac{1}{2}\frac{\partial C_g}{\partial x}(V_g - V_{sd})^2 \quad (1)$$

Where $\frac{\partial C_g}{\partial x}$ is the spatial derivative of the gate capacitance, V_g is the static voltage applied to the local gate electrode and V_{sd} is the AC voltage applied between the source and drain electrodes of the device.

This force F is the sum of the electrostatic DC force F_{es} and the AC driving forceF_{drive}, described respectively as

$$|F_{es}| = \frac{\partial C_g}{\partial x}V_g^2 \quad (2)$$

$$|F_{drive}| \sim \frac{\partial C_g}{\partial x}V_g V_{sd} \quad (3)$$

Ti_3C_2Tx MXene membrane is subjected to the force of the gate voltage causing the depression of the membrane, and this deformation process generates piezoelectric polarization charges, which affects the height of the Schottky barrier in Au-MXene-Au, and thus the observed I-V curve shows an asymmetric special modulation phenomenon.

6. Analysis of the intensity of piezoelectric effects in each mode.

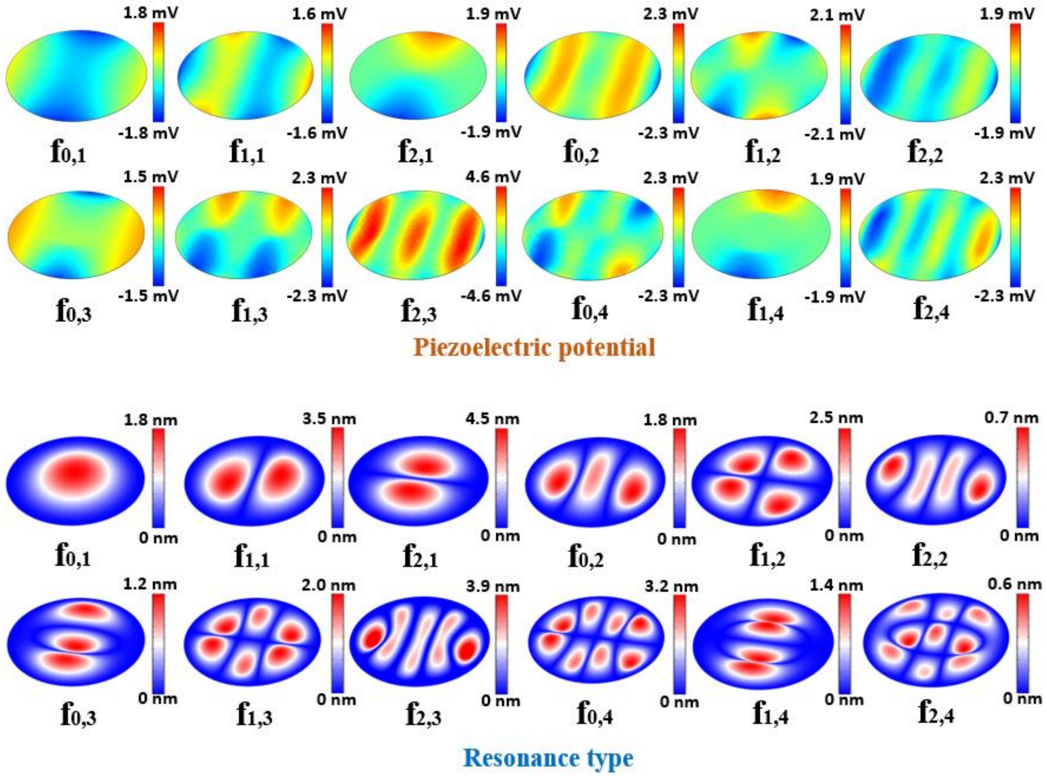

Figure S5 Response current corresponding to different resonant modes.

During the high amplitude vibration, the inner layer will generate a stronger piezoelectric response signal that leaks in the metal-Ti_3C_2Tx MXene contact area, creating a more significant piezoelectric charge modulation effect, and this piezoelectric signal from the inner layer will result in a significant enhancement of the vibration signal during the high amplitude vibration. As a good conductor, the resonance effect results in a piezoelectric output whose strength depends on the overall deformation strength, and the piezoelectric signal generated by low strain in low-order modes is neutralized by the AC signal and free electrons thus making it

difficult to generate a measurable signal. The piezoelectric signal will obtain significant signal enhancement in complex resonance modes, leading to differentiation of the inter-modal output. As shown in **Figure S5**, the strain limit of ~1.05% is reached in the $f_{2,3}$ mode, achieving a piezoelectric potential of up to 4.6 mV.

7. The relation between the amplitude of deformation and piezoelectric response.

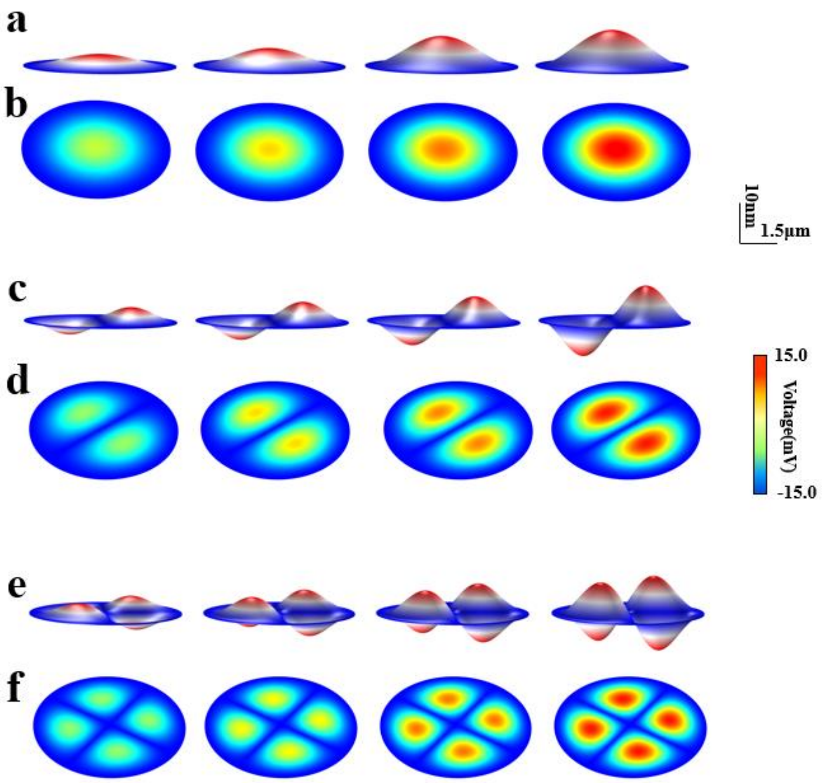

Figure S6 a, **c**, **e** The modes with different amplitudes of deformation. **b**, **d**, **f** Piezoelectric response potential distribution of the modes with different deformation.

The gate voltage exerts a drag force on the Ti_3C_2Tx MXene membrane, which can be described as:

$$F = -\frac{1}{2}\frac{\partial C_g}{\partial x}(V_g - V_{sd})^2 \qquad (4)$$

Where $\frac{\partial C_g}{\partial x}$ is the spatial derivative of the gate capacitance, V_g is the static voltage applied to the local gate electrode, and V_{sd} is the AC voltage applied between the source and drain electrodes of the device.

With the source-drain voltage determined, the change in gate voltage directly affects the force applied to the monolayer Ti_3C_2Tx MXene, thus producing differences in the deformation amplitude of the monolayer Ti_3C_2Tx MXene. Corresponding to the different deformation amplitude, higher internal deformation will produce a more intense piezoelectric polarization, resulting in a significant difference in the resonant response signal.

8. The frequency response and the equation of motion of resonators driven by Duffing damping. [8-12]

The nonlinear intrinsic strain-stress relation is arisen by increasing deformation, which could be explained by the nonlinear effects depending on amplitude in the equation of motion.

The resonators operate in the linear regime with low driving force (the driving force that leads to the mechanical vibrations of the device), the motional amplitude x results in the appearance of nonlinear response by increasing the driving force. In this case, due to the x being increased, the nonlinear restoring force (ax^3) and nonlinear damping $(\eta x^2\dot{x})$ are supposed to be concerned. the equation of motion, known as the van der Pol-Duffing Equation, is described as:

$$\ddot{x} + \frac{k}{m}x + \left(\frac{\gamma}{m} + \frac{\eta}{m}x^2\right)\dot{x} + \frac{\alpha}{m}x^3 = \frac{F_0}{m}cos(\omega t) \qquad (5)$$

where x is the motional amplitude, k is the effective spring constant, γ is the linear damping coefficient, η is linear damping coefficient, α is the Duffing force coefficient, F_0 is the amplitude of the driving force, m is the resonator mass, ω is the driving frequency and t is the time. Lifshitz et. al. has calculated the solution of the van der Pol-Duffing equation in the limit of week linear damping (Q smaller than 1000) by using secular perturbation theory, the expressions of the steady-state are obtained, which are described by

$$x_0^2(\omega) = \frac{\left(\frac{F_0}{2m\omega_0^2}\right)^2}{\left(\frac{\omega-\omega_0}{\omega_0} - \frac{3\alpha}{8m\omega_0^2}x^2(\omega)\right)^2 + \left(\frac{1}{2Q} + \frac{\eta}{8m\omega_0}x_0^2(\omega)\right)^2} \qquad (6)$$

and

$$\tan(\emptyset) = \frac{\frac{\gamma}{2} + \frac{\eta}{8}x_0^2(\omega)}{m(\omega-\omega_0) - \frac{3\alpha}{8\omega_0^2}x^2(\omega)} \qquad (7)$$

Here, x_0 is the motional amplitude and the $\emptyset$ is the phase. By inserting $\alpha = \eta = 0$, the above expression is reduced to the response of a linear harmonic oscillator.

9. Dynamic behavior of the Ti_3C_2Tx MXene resonator by finite element modeling and analysis.[8]

Finite element models are built by COMSOL 5.5. The electromechanical Multiphysics package combines the "solid mechanics", "electrostatics", and "dynamic grid function" to simulate the deformation of electrostatic driven structures, which allows the investigation of the dynamic mechanical behavior of MXene membranes, including the modulation of local gate voltage on the resonant frequency with different mass densities, built-in strains, and electrostatic driving forces.

The MXene resonator is modeled as an isotropic linear elastic two-dimensional membrane suspended over an air cavity. The material parameters of MXene are as follows: Poisson's ratio $\upsilon = 0.227$ and Young's modulus $E = 330\text{GPa}$. The diameter of the membrane is 3000 nm and the thickness is 1nm. The Air cavity is modeled as a linear elastic dielectric, which can deform in the direction perpendicular to the membrane. And the relative dielectric constant of the non-solid air material is 1. The membrane is tightened on the hole, which is the mechanical boundary condition of the model. A voltage terminal applied a direct current (DC) gate voltage is connected to the lower boundary of the dielectric material. Small signal perturbation voltage is applied between the clamped edges of the membrane to simulate the drain-source

alternating current (AC). Through statics study, the static vertical deflection and the static strain distribution of membrane determined by the electrostatic DC force. The characteristic frequency of the membrane is determined by solving the model with a stationary analysis study, where the resonant frequency corresponds to the determined characteristic frequency. The effect of gate voltage on tuning the resonant frequency is achieved by giving a range of gate voltages to solve the model with running sweeps. The relationship between resonant frequency and the gate voltage is obtained and the results are presented in **Figure S7a**. The resonant frequencies with the same gate voltage at different mass densities are recorded by giving a range of mass densities and solving the model while running sweeps over, the results are presented in **Figure S7b**. **Figure S7c** shows the shape of the vibrations of the first three orders.

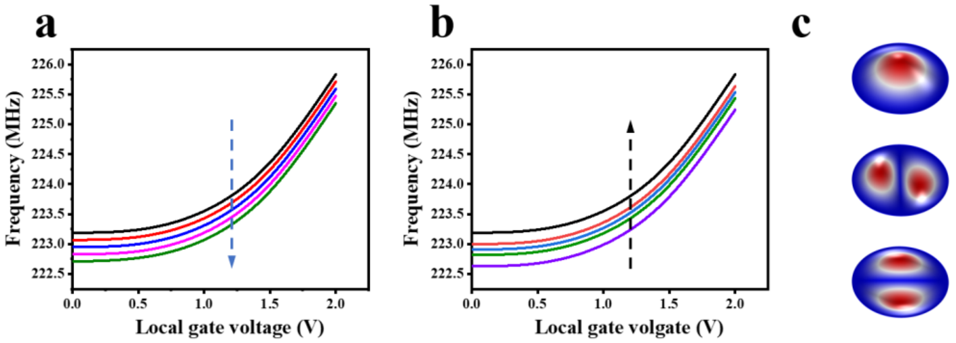

Figure S7 a The built-in strain increases followed by the arrowhead. The higher resonance frequency occurs at larger built-in strain because the contribution of electrostatic induced strain is smaller than that of the

built-in strain. **b** The mass density increases followed by the arrowhead. Resonators with higher mass densities correspond to lower resonant frequencies. And the resonator with high mass density possesses low sensitivity to the applied gate voltage. **c**, The vibrations shape of the first three orders.

10. Resonance frequency as a function of the layer number of Ti_3C_2Tx MXene resonators.

For different layers of Ti_3C_2Tx MXene nanoelectromechanical resonators, the resonant frequencies exhibit variability. An ANOVA Tukey test is used to analyze the relationship between the number of layers and the resonant frequency. The specific values of Mean of Squares Group (MSG) and Mean of Squares Error (MSE) can be calculated, so $F = \frac{MSG}{MSE} = 3.62$. It can be seen from the probability statistics table of F that $F_{0.05}(6,25) = 2.51$, so $F \geq F_{0.05}(6,25)$. With the significance level of 0.05, the frequency exhibits clear independence from the number of MXene layers as the statistical values in **Figure S8**.

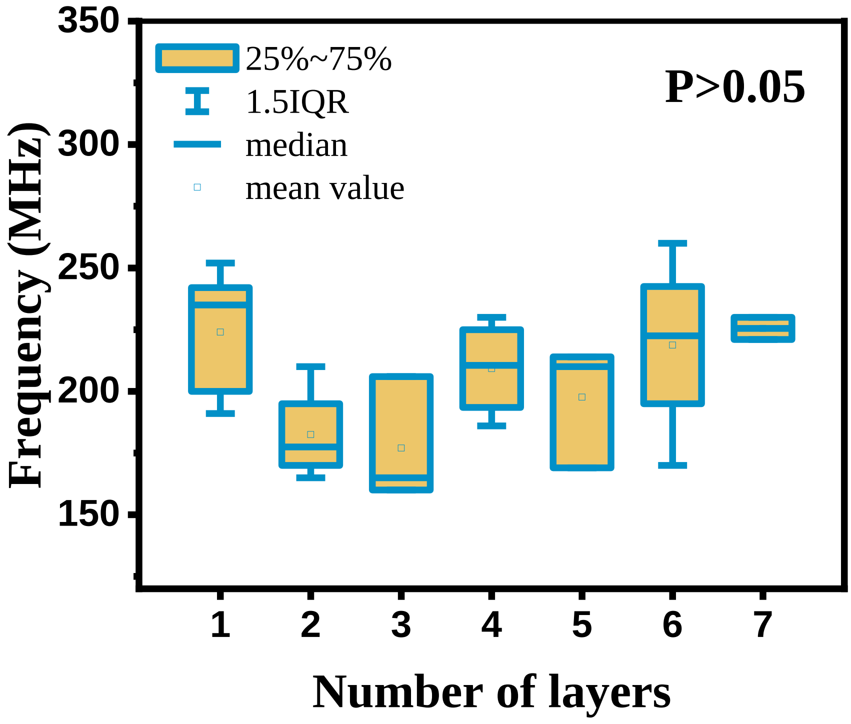

Figure S8 Measured characteristic resonance frequency as a function of

the layer number of Ti_3C_2Tx MXene resonators.

The statistical results show that the resonant frequencies of the Ti_3C_2Tx MXene resonators decrease at the beginning and then increase with the larger layer number. This phenomenon is mainly caused by the variation of the pretension and the overall Young's modulus of the layers.

11. Young's modulus model and variation law of single atomic layer of Ti_3C_2Tx MXene.

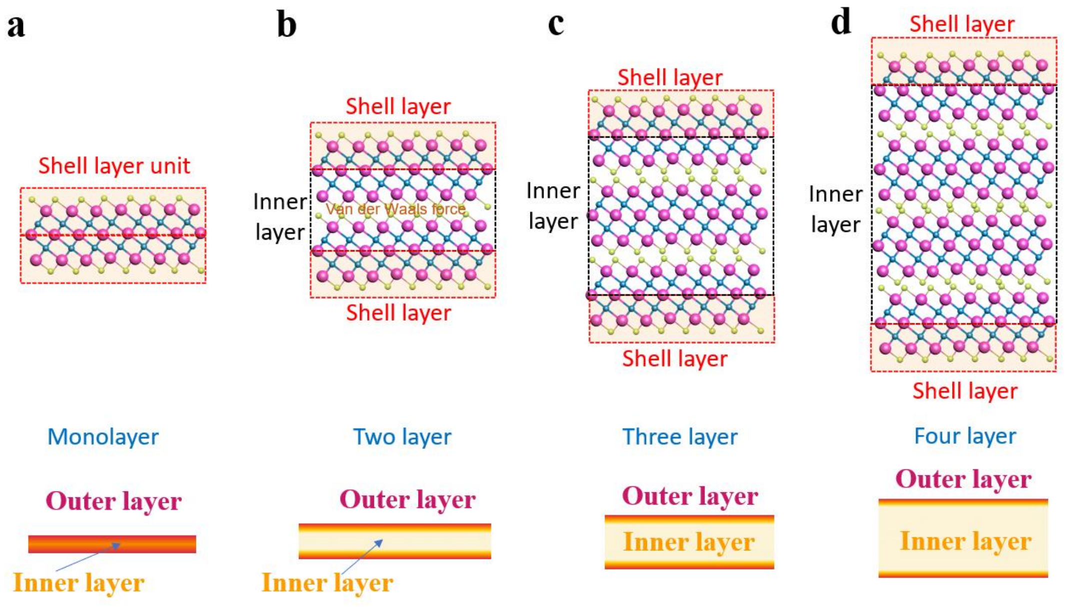

Figure S9 a The side view of a monolayer Ti_3C_2Tx MXene atomic structure and the corresponding Young's modulus model. **b** The side view of the three layers MXene atomic structure and the corresponding Young's modulus model.

The distinctive functional groups of Ti_3C_2Tx MXene have a certain influence on Young's modulus as shown in **Figure S9a**. As a single-atomic layer structure, the surface effect becomes significant due to the large surface-to-volume ratio of Ti_3C_2Tx MXene. The resonance deformation process exhibits a more complex high-frequency vibrational deformation compared to the nanoindentation experiments, where the stress and strain on the surface are greatest due to the large distance

between the surface and the neutral layer. And the significant relaxation generated by the contraction of the bond length of the surface functional groups may have a significant effect on the surface elastic properties since the elastic constants of the crystal are indeed sensitive to the interatomic distance. As shown in **Figure S9**, the variation of interlayer interaction during Ti_3C_2Tx MXene stacking is demonstrated. The monolayer Ti_3C_2Tx MXene is divided into effective repeating units of Tx-Ti-C, when the influence of external functional groups on the overall Young's modulus dominates the factor. As shown in **Figure S9b**, **c**, and **d**, the inner layer of interlayer interconnection with interlayer van der Waals forces dominates the overall mechanical properties of the film and Young's modulus tends to be stable with the stacking of layers.

12. Adsorption model of the molecule on Ti_3C_2Tx MXene.[13]

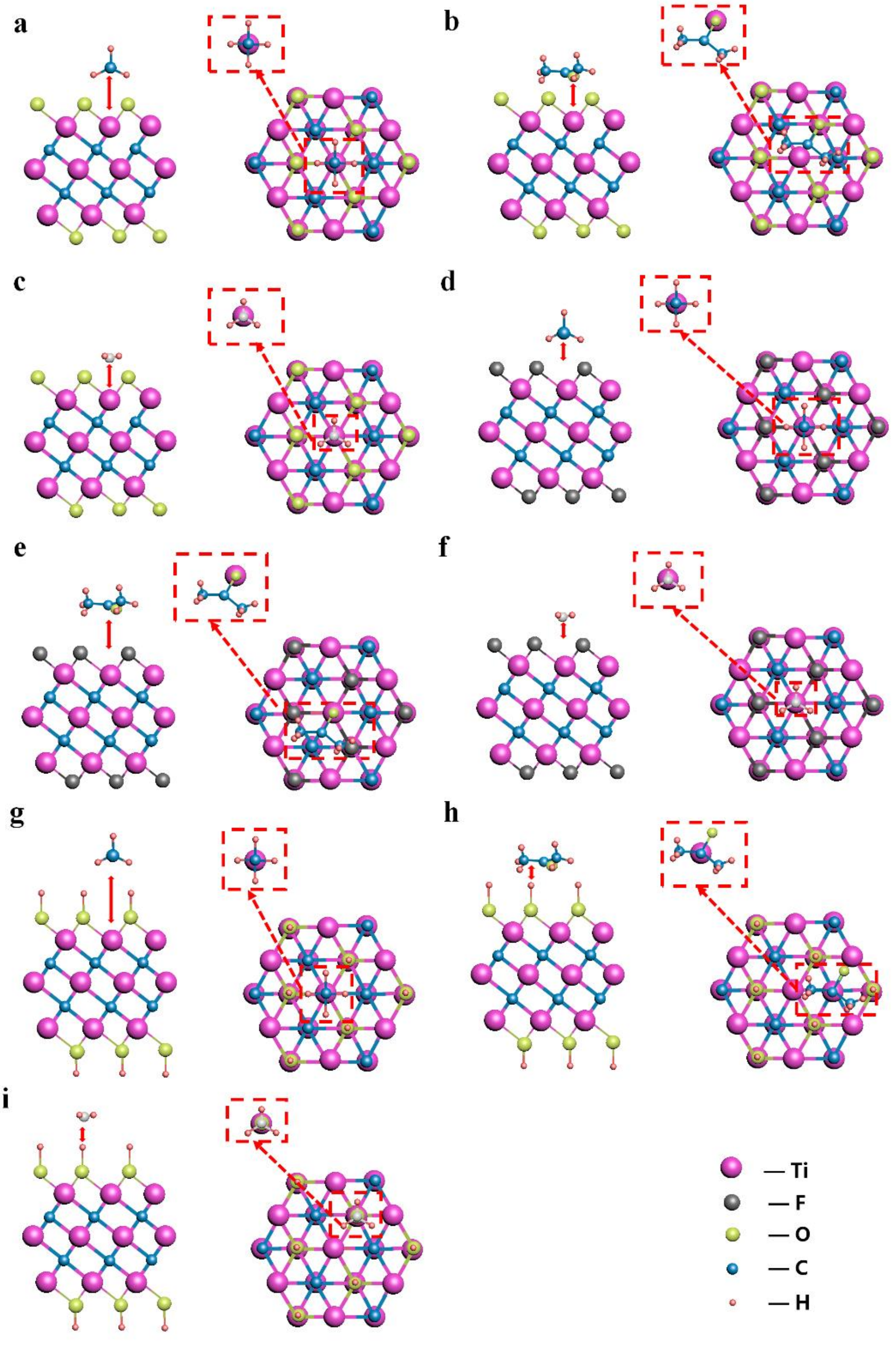

Figure S10 Minimum energy (primary) adsorption configurations for acetone, ammonia, and methane. Side view and top view of the most favorable configurations for **a**, methane, **b**, acetone, **c**, ammonia on Ti_3C_2Tx. Side view and top view of the most favorable configurations for **d**, methane, **e**, acetone, **f**, ammonia on Ti_3C_2Tx. **g-i,** Side view, and top view of the most favorable configurations for **g**, methane, **h**, acetone, **i**, ammonia on Ti_3C_2Tx.

Ti_3C_2Tx MXene exhibits good sensing properties for both organic and inorganic gas molecules, and the functional groups provide diverse molecular adsorption modes.

Table S1. DFT calculation results for acetone, ammonia, methane, nitrogen, and hydrogen molecules.[13,14]

Gas	Material	h (Å)	Ea (kJ/mol)
**Acetone**	$Ti_3C_2(OH)_2$	1.23	-74.67
	$Ti_3C_2O_2$	2.59	-30.59
	$Ti_3C_2F_2$	2.43	-30.01
**Ammonia**	$Ti_3C_2(OH)_2$	1.81	-47.41
	$Ti_3C_2O_2$	1.69	-34.25
	$Ti_3C_2F_2$	2.21	-16.60

**Methane**	$Ti_3C_2(OH)_2$	—	-7.048
	$Ti_3C_2O_2$		-7.860
	$Ti_3C_2F_2$		-6.444
**Nitrogen**	$Ti_3C_2(OH)_2$	—	-5.274
	$Ti_3C_2O_2$		-5.850
	$Ti_3C_2F_2$		-4.860
**Hydrogen**	$Ti_3C_2(OH)_2$	—	-1.975
	$Ti_3C_2O_2$		-2.307
	$Ti_3C_2F_2$		-1.825
**Acetone**	BP	2.98	-31.67
	Graphene	3.60	-35.43
	MoS_2	3.43	-21.64
**Ammonia**	BP	2.83	-21.43
	Graphene	2.94	-12.69
	MoS_2	2.75	-15.60
**Ammonia**	Ti_2CO_2	2.35	-34.65
**Methane**	Ti_2CO_2	3.06	-10.60
**Nitrogen**	Ti_2CO_2	3.71	-11.56
**Hydrogen**	Ti_2CO_2	2.76	-4.82

Where h and Ea indicate the vertical distance and binding energy between the gas molecules and 2D materials at adsorption equilibrium. As shown

in **Table S1**, the binding energy of the -OH functional group of MXene (Ti_3C_2Tx) to gas (NH_3 and acetone) is obviously higher than the gas adsorption energy Ea of other 2D materials (BP, graphene, and MoS_2), and the vertical distance h between gas molecules and 2D materials in equilibrium is relatively short. Therefore, MXene (Ti_3C_2Tx) has a stronger adsorption capacity for gas and a more stable adsorption effect. In consequence, as a gas molecular monitoring device, the lowest limit of gas detection concentration of MXene is far lower than that of other 2D materials. At the same time, MXene ($Ti_3C_2(OH)_2$) has high binding energy for ammonia and acetone, and MXene (Ti_3C_2Tx) has high selectivity for ammonia and acetone because -OH functional groups can form hydrogen bonds with ammonia and acetone.

13. Evaluation and comparison of the adsorption capacity of MXene molecules.[15]

The adsorption energy of low molecular-mass gas molecules on the 2D material surface can be expressed by interaction potential energy U:

$$U = \frac{2E_a d_0^6}{d^6} - \frac{2E_a d_0^{12}}{d^{12}} \quad (8)$$

Where the adsorption energy Ea is between gas molecules and the 2D material surface, d is the distance between gas molecules and the MXene surface, and d_0 is the distance between gas molecules and the surface of the 2D material when the gas adsorption equilibrium is reached.

Nonlinear stiffness spring is used to describe the interaction between gas molecules and the 2D material surface, and the interaction force F between gas molecules and the 2D material can be described as:

$$F = \frac{dU}{dd} \quad (9)$$

As shown in **Table S2** and **Table S3**, during the adsorbing process of ammonia and acetone molecules, the interaction force F between the surface of MXene and the gas molecule is far greater than that between other 2D materials (BP/graphene/ MoS_2) and the gas molecule, which indicates that MXene has a much higher selectivity and a more stable adsorption effect for ammonia and acetone.

Table S2. The interaction forces F between Acetone, Ammonia, and surfaces of MXene during adsorption equilibrium.

Material	Gas	F(10^{-10}N)
**$Ti_3C_2(OH)_2$**	Acetone	-121.0
	Ammonia	-52.2
**$Ti_3C_2O_2$**	Acetone	-23.5
	Ammonia	-40.4
**$Ti_3C_2F_2$**	Acetone	-24.6
	Ammonia	-15.0
**Ti_2CO_2**	Ammonia	-29.4

Table S3. The interaction forces F between Acetone, Ammonia, and other surfaces of 2D materials during adsorption equilibrium.

Material	Gas	F(10^{-10}N)
**BP**	Acetone	-21.2
	Ammonia	-15.1
**Graphene**	Acetone	-19.6
	Ammonia	-8.6
**MoS_2**	Acetone	-12.6
	Ammonia	-11.3

14. Analysis of the attachment mode of the target molecules.

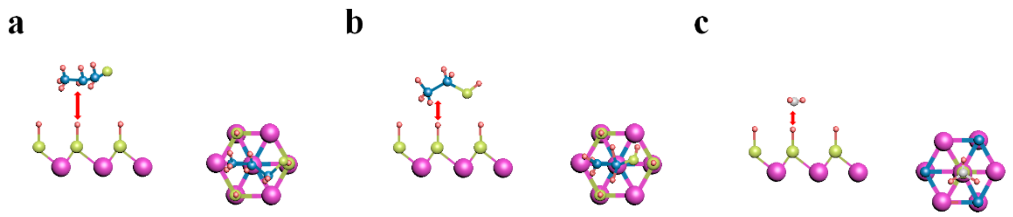

Figure S11 Side view and top view of the most favorable configurations for a, propanal, b, ethanol, c, ammonia on $Ti_3C_2OH_2$.

As shown in **Figure S11**, when the adsorption equilibrium is achieved, the vertical distance between gas molecules and $Ti_3C_2OH_2$ is short because the presence of hydrogen bonds makes the adsorption energies between gas molecules (propanal, ethanol, ammonia) and $Ti_3C_2OH_2$ large. However, the vertical distance between the $Ti_3C_2OH_2$ and the three gas molecules is different due to the different molecular mass, which leads to a great difference in the minimum energy adsorption sites of the three gas molecules on $Ti_3C_2OH_2$. The minimum energy adsorption sites of propanal and ethanol are located on the Ti sites that are the pivot of the three functional groups -OH, while ammonia is directly adsorbent vertically above the functional group -OH.

15. The mass resolution and dynamic range of the MXene resonators.[16]

The linear dynamic range of resonators could be determined, due to the presence of nonlinear behavior in MXene NEMS resonators. When nonlinear behavior occurs, the critical mechanical amplitude can be obtained from the equation:

$$a_c = \sqrt{\frac{8\sqrt{3}}{9k^2Q}} \qquad (10)$$

Where a_c is critical amplitude, k is the duffing-type nonlinear coefficient.

For a circular Ti_3C_2Tx MXene membrane, the duffing-type nonlinear coefficient k can be expressed as

$$k^2 = \frac{13+21v-4v^2}{30(1+v)r^2\varepsilon} \qquad (11)$$

Where v is the Poisson's ratio of the Ti_3C_2Tx MXene, r is the radius of the membrane, and ε is the initial strain.

The dynamic range (DR) for linear operation of the monolayer Ti_3C_2Tx MXene resonator is then defined by

$$DR \equiv 20\log(\frac{0.745a_c}{\sqrt{2S_z\Delta f}}) \qquad (12)$$

where Δf is the measurement bandwidth, Δf is the measurement bandwidth and S is the spectral density of the thermomechanical noise at a resonant frequency given by

$$\mathrm{S = 4k_BTQ/M_{eff}\omega_m^3} \qquad (13)$$

Here k_B is the Boltzmann constant, T is the temperature and M_{eff} is the effective mass. Furthermore, we can use the dynamic range obtained

to infer the mass resolution of the resonator:

$$\delta m \approx \frac{2M_{eff}}{Q} \times 10^{-\frac{DR(dB)}{20}} \qquad (14)$$

16. Displacement sensitivity calibration.[9]

It is possible to relate the amplitude of the thermomechanical motion to the measured noise level in the spectrum, and the spectral density of the thermomechanical motion in the frequency domain for any resonant mode is

$$S_{x,th}^{\frac{1}{2}}(\omega) = \sqrt{\frac{4\omega_n k_B T}{QM_{eff}} \cdot \frac{1}{(\omega_m^2-\omega^2)^2+(\omega_m\omega/Q)^2}} \qquad (15)$$

Further, when $\omega = \omega_m$, it can be expressed as

$$S_{x,th}^{\frac{1}{2}}(\omega_m) = \sqrt{\frac{4k_B TQ}{M_{eff}\omega_m^3}} \qquad (16)$$

Here k_B is the Boltzmann constant, T is the temperature and M_{eff} is the effective mass, Q is the quality factor, and ω_m is angular resonance frequency.

The effective mass can be deduced from the resonant cavity diameter

$$M_{eff} = \frac{1}{8}\pi d^2 \rho h \qquad (17)$$

Where d is the diameter of the resonant cavity, ρ is the density of Ti_3C_2Tx MXene (3.41 $\frac{g}{cm^3}$), and h is the thickness of Ti_3C_2Tx MXene membrane.

The noise process is assumed to be uncorrelated, so there is

$$S_{V,total}^{\frac{1}{2}} = \sqrt{S_{V,th} + S_{V,sys}} \qquad (18)$$

Where $S_{V,th}^{\frac{1}{2}}$ is the electronic-domain noise spectral density induced by

the thermomechanical motion.

$$R = \frac{S_{V,th}^{\frac{1}{2}}}{S_{x,th}^{\frac{1}{2}}} \tag{19}$$

Where $S_{V,th}^{\frac{1}{2}}$ is the voltage noise floor of the measurement system. Here, $S_{V,sys}^{\frac{1}{2}} \approx 0.1 - 1.5\,\mu\,VHz^{-\frac{1}{2}}$ in 10-250 MHz frequency range, which sets the off-resonance background ($S_{V,total}^{\frac{1}{2}} \approx S_{V,sys}^{\frac{1}{2}}$ when $\omega \neq \omega_m$).

The displacement sensitivity of the test system can be defined as

$$S_{V,sys}^{\frac{1}{2}} = \frac{1}{R} S_{V,total}^{\frac{1}{2}}(\omega \neq \omega_m) = \frac{S_{x,th}^{\frac{1}{2}}(\omega=\omega_m)}{S_{V,th}^{\frac{1}{2}}(\omega=\omega_m)} S_{V,total}^{\frac{1}{2}}(\omega \neq \omega_m) = \frac{S_{x,th}^{\frac{1}{2}}(\omega=\omega_m)}{\sqrt{S_{V,total(\omega=\omega_m)} - S_{V,sys(\omega=\omega_m)}}} S_{V,total}^{\frac{1}{2}}(\omega \neq \omega_m) \tag{20}$$

For a typical measurement system, $S_{V,sys}^{\frac{1}{2}}$ is kept stable and $S_{V,total}^{\frac{1}{2}}(\omega = \omega_m) = 1.0\,\mu\,VHz^{-\frac{1}{2}}$ and $S_{V,sys}^{\frac{1}{2}}(\omega = \omega_m) = 0.5\,\mu\,VHz^{-\frac{1}{2}}$ is used here. And $S_{x,sys}^{\frac{1}{2}}$ can be obtained to describe the sensitivity of the displacement domain provided by the experimental setup in this experiment

$$S_{x,sys}^{\frac{1}{2}} = \frac{9.664\times10^{-15}\times0.5}{\sqrt{1.0^2-0.5^2}} = 5.427\frac{fm}{\sqrt{Hz}} \tag{21}$$

17. Comparison of thermodynamic noise spectral densities and mass resolution of 2D nanomechanical resonators.

Material	$S_{x,th}^{\frac{1}{2}}$ $(\frac{fm}{\sqrt{Hz}})$	δ_m (ag)	Ref.
**MXene**	9.441	0.22	Our work
**Graphene**	260	1.41	[17]
**BP**	30	3	[18]
**MoS_2**	23	2.5	[19]
**Si**	16	7×10^6	[20]
**SiC**	14	3.1	[9]

18. Frequency Allan deviation at the half maximum amplitude of the resonance.

The noise is measured at half maximum amplitude of the resonance as shown in **Figure S11a**, it is the point of maximum derivative $\frac{dI}{df}$ where the mechanical frequency noise is fully transduced into current noise. By extension, measuring the noise at this peculiar point on a long time scale (4700 s) defines the frequency Allan deviation as shown in **Figure S11b**.

We calculate the Allan deviation σ_A as

$$\sigma_A^2 = \frac{1}{2(n-1)} \sum_{i=1}^{n-1} (y(i) - y(i-1))^2 \qquad (22)$$

where $y(i)$ and $y(i-1)$ are two subsequent measurements of current noise as shown in **Figure S11a**. n is the number of averaged current measurements.

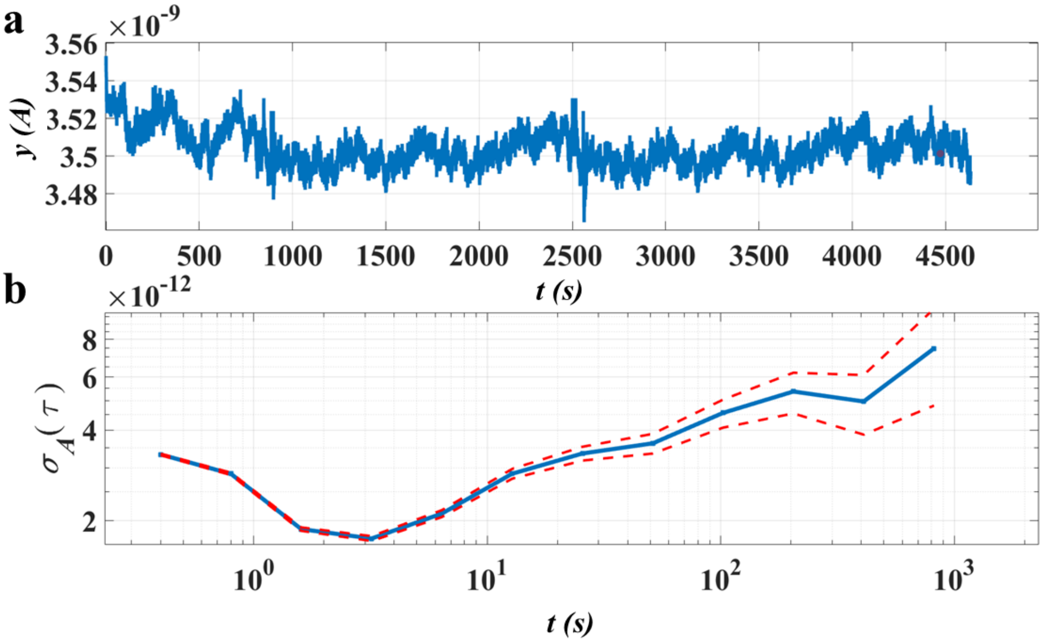

Figure S12 a, Noise current measured at half the maximum resonance amplitude. **b**. Allan deviation of the noise current.

Reference

[1] A. Lipatov, H. Lu, M. Alhabeb, B. Anasori, A. Gruverman, Y. Gogotsi, A. Sinitskii, *SCIENCE ADVANCES* **2018**, *4*.

[2] M. Alhabeb, K. Maleski, B. Anasori, P. Lelyukh, L. Clark, S. Sin, Y. Gogotsi, *Chemistry of Materials* **2017**, *29*, 7633.

[3] D. Tan, N. Sun, L. Chen, J. Bu, C. Jiang, *ACS Applied Nano Materials* **2021**.

[4] D. Tan, C. Jiang, N. Sun, J. Huang, Z. Zhang, Q. Zhang, J. Bu, S. Bi, Q. Guo, J. Song, *Nano Energy* **2021**, *90*, 106528.

[5] W. Ma, J. Lu, B. Wan, D. Peng, Q. Xu, G. Hu, Y. Peng, C. Pan, Z. L. Wang, *ADVANCED MATERIALS* **2020**, *32*.

[6] Z. L. Wang, *NANO TODAY* **2010**, *5*, 540.

[7] A. Castellanos-Gomez, R. van Leeuwen, M. Buscema, H. S. J. van der Zant, G. A. Steele, W. J. Venstra, *Advanced Materials* **2013**, *25*, 6719.

[8] S. Manzeli, D. Dumcenco, G. Migliato Marega, A. Kis, *Nature Communications* **2019**, *10*.

[9] Z. Wang, J. Lee, P. X. L. Feng, *Nature Communications* **2014**, *5*.

[10] J. P. Mathew, R. N. Patel, A. Borah, R. Vijay, M. M. Deshmukh, *Nature Nanotechnology* **2016**, *11*, 747.

[11] C. Chen, S. Lee, V. V. Deshpande, G. Lee, M. Lekas, K. Shepard, J. Hone, *Nature Nanotechnology* **2013**, *8*, 923.

[12] A. Eichler, J. Moser, J. Chaste, M. Zdrojek, I. Wilson-Rae, A. Bachtold, *Nature Nanotechnology* **2011**, *6*, 339.

[13] S. J. Kim, H. Koh, C. E. Ren, O. Kwon, K. Maleski, S. Cho, B. Anasori, C. Kim, Y. Choi, J. Kim, Y. Gogotsi, H. Jung, *ACS Nano* **2018**, *12*, 986.

[14] X. Li, H. Cui, R. Zhang, S. Li, *Vacuum* **2020**, *179*, 109574.

[15] J. Sun, M. Muruganathan, H. Mizuta, *Science Advances* **2016**, *2*.

[16] Aaryashree, P. V. Shinde, A. Kumar, D. J. Late, C. S. Rout, *Journal of materials chemistry. C, Materials for optical and electronic devices* **2021**, *9*, 3773.

[17] M. Kumar, H. Bhaskaran, *Nano Letters* **2015**, *15*, 2562.

[18] Z. Wang, H. Jia, X. Zheng, R. Yang, Z. Wang, G. J. Ye, X. H. Chen, J. Shan, P. X. L. Feng, *Nanoscale* **2015**, *7*, 877.

[19] C. Jiang, Q. Li, J. Huang, S. Bi, R. Ji, Q. Guo, *ACS Appl Mater Interfaces* **2020**, *12*, 41991.

[20] R. M. R. Pinto, P. Brito, V. Chu, J. P. Conde, *Journal of Microelectromechanical Systems* **2019**, *28*, 390.
